# Supramolecular Gel‐Derived Highly Efficient Bifunctional Catalysts for Omnidirectionally Stretchable Zn–Air Batteries with Extreme Environmental Adaptability

**DOI:** 10.1002/advs.202200753

**Published:** 2022-05-06

**Authors:** Junpeng Liu, Mengke Wang, Chaonan Gu, Jingjing Li, Yujia Liang, Hai Wang, Yihan Cui, Chun‐Sen Liu

**Affiliations:** ^1^ Henan Provincial Key Laboratory of Surface & Interface Science Zhengzhou University of Light Industry Zhengzhou 450002 China; ^2^ School of Chemistry and Chemical Engineering Henan University of Technology Zhengzhou 450001 China

**Keywords:** mechanical and environmental adaptability, omnidirectionally stretchable battery, ultra‐low temperature tolerance, waterproofness

## Abstract

Most existing stretchable batteries can generally only be stretched uniaxially and suffer from poor mechanical and electrochemical robustness to withstand extreme mechanical and environmental challenges. A highly efficient bifunctional electrocatalyst is herein developed via the unique self‐templated conversion of a guanosine‐based supramolecular hydrogel and presents a fully integrated design strategy to successfully fabricate an omnidirectionally stretchable and extremely environment‐adaptable Zn–air battery (ZAB) through the synergistic engineering of active materials and device architecture. The electrocatalyst demonstrates a very low reversible overpotential of only 0.68 V for oxygen reduction/evolution reactions (ORR/OER). This ZAB exhibits superior omnidirectional stretchability with a full‐cell areal strain of >1000% and excellent durability, withstanding more than 10 000 stretching cycles. Promisingly, without any additional pre‐treatment, the ZAB exhibits outstanding ultra‐low temperature tolerance (down to −60 °C) and superior waterproofness, withstanding continuous water rinsing (>5 h) and immersion (>3 h). The present work offers a promising strategy for the design of omnidirectionally stretchable and high‐performance energy storage devices for future on‐skin wearable applications.

## Graphical abstract

Most existing stretchable batteries can generally only be stretched uniaxially. Herein, a fully integrated design strategy is presented to successfully fabricate an omnidirectionally stretchable Zn–air battery (ZAB) with a full‐cell areal strain of >1000%. This ZAB also exhibited excellent mechanical and electrochemical robustness, ultra‐low temperature (−60 °C), and underwater immersion.

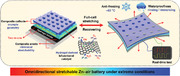

## 1 Introduction

Over the past decades, flexible batteries capable of bending and folding have been developed with enhanced performance, chemistry, and fabrication methods for powering typical wearable devices such as smartwatches and headbands.^[^ ^1^, ^2^, ^3^, ^4^, ^5^, ^6^ ^]^ The next generation of on‐skin wearables requires highly stretchable batteries to accommodate both the strain and flexure of human skin.^[^ ^7^, ^8^, ^9^ ^]^ However, imparting high stretchability to conventionally rigid batteries is technically much more challenging than achieving simple flexibility.^[^ ^8^, ^10^ ^]^ At present, stretchable batteries are fabricated with special electrode/battery configurations such as buckled wavy or porous structures, rigid islands, or folding and fiber‐like structures.^[^ ^11^, ^12^, ^13^, ^14^, ^15^ ^]^ These micro/nano‐scale engineering strategies usually provide satisfactory deformation capability. However, only predefined deformation patterns (generally uniaxially stretching) can be achieved due to their highly complicated battery geometries, which cause difficulties with device integration and next‐generation on‐skin applications.^[^ ^12^ ^]^ Devices based on inherently stretchable components are expected to be isotropically stretchable, but these devices currently suffer from low strain capability and low active material loading.^[^ ^16^, ^17^, ^18^, ^19^, ^20^ ^]^ Therefore, achieving high‐performance stretchable batteries with excellent omnidirectional strain capability is still a significant challenge.

Existing stretchable batteries also suffer from poor mechanical and electrochemical robustness to withstand harsh mechanical and environmental challenges such as extreme compression, ultra‐low temperatures, and underwater immersion.^[^ ^21^, ^22^, ^23^ ^]^ In this regard, the design of active materials is crucial for achieving superior electrochemical performance, especially under harsh conditions.^[^ ^24^, ^25^, ^26^ ^]^ Recently, nanostructured gel materials have become an attractive material platform for energy‐related applications due to their hierarchical porous 3D framework structure, high compositional tunability, ease of synthesis, and easy functionalization.^[^ ^27^, ^28^, ^29^, ^30^ ^]^ However, due to their poor crystallinity and structural instability at high temperatures, the application of supramolecular gels for fabricating highly efficient bifunctional electrocatalysts capable of surviving extreme mechanical and environmental conditions is highly challenging and has never been reported. Therefore, a significant amount of work is still required on both the active material and device levels to rationally design highly stretchable batteries that simultaneously exhibit high electrochemical performance, remarkable isotropic stretchability, and extreme environmental adaptability.

**Scheme 1:**
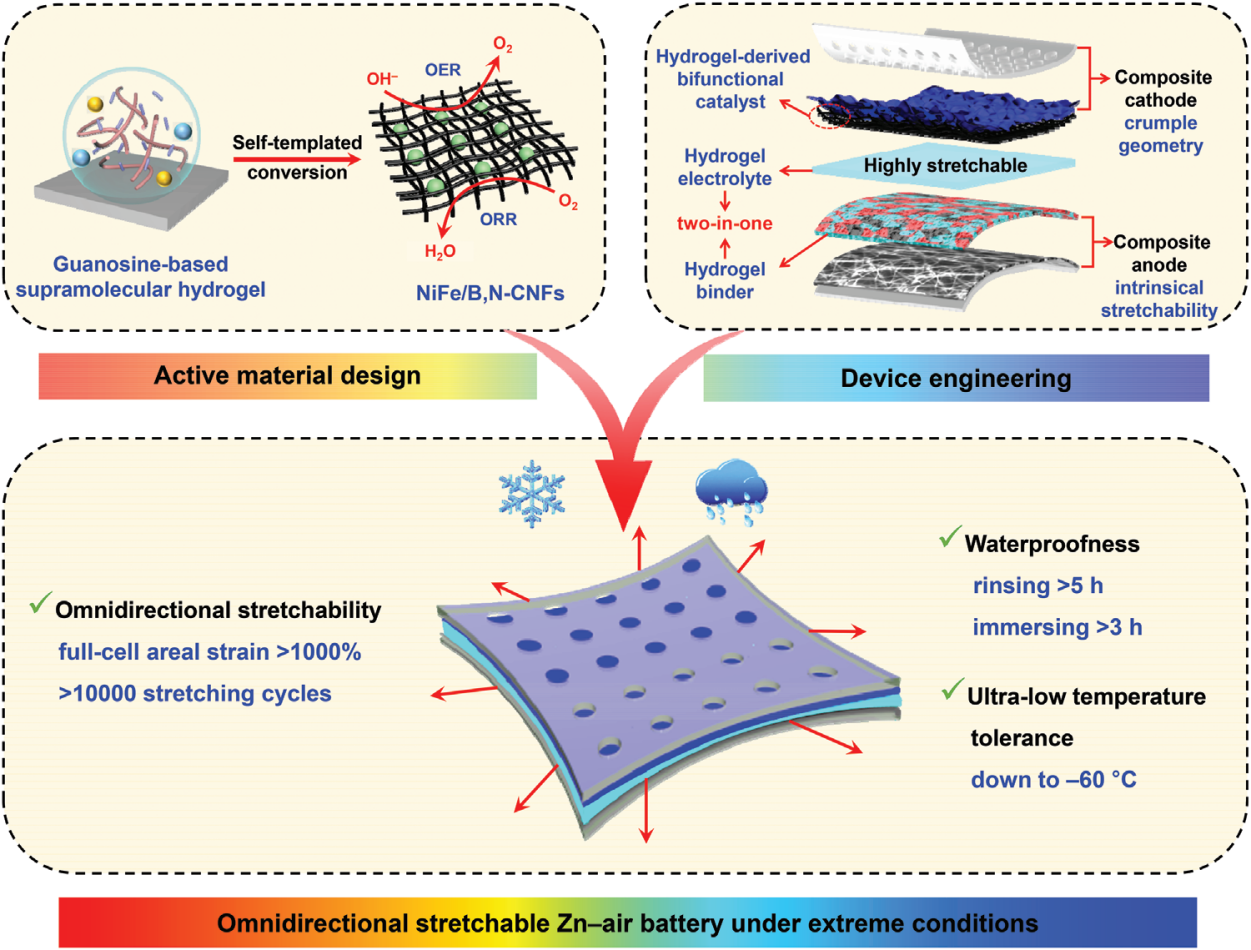
Design strategy of omnidirectional stretchable Zn–air battery through the synergistic engineering of active materials and device architecture. Upper left: the self‐templated conversion of a GSMG into a bifunctional NiFe/B,N‐CNFs electrocatalyst. Upper right: the device architecture for the creation of an omnidirectionally stretchable ZAB by using the same guanosine‐based hydrogel as both the electrolyte and electrode binders. Bottom: the outstanding performance of the omnidirectional stretchable ZAB capable of withstanding extreme environmental challenges.

Herein, we present an omnidirectional stretchable Zn–air battery (ZAB) with high electrochemical performance and extreme environmental adaptability through the synergistic engineering of active materials and device architecture (**Scheme** **1**). First, a highly efficient bifunctional electrocatalyst consisting of NiFe alloy nanoparticles embedded in B,N‐doped carbon nanofibers (NiFe/B,N‐CNFs) were synthesized via the unique self‐templated conversion of a guanosine‐based supramolecular hydrogel (GSMG). This electrocatalyst demonstrated a very low reversible overpotential of only 0.68 V for oxygen reduction/evolution reactions (ORR/OER). Subsequently, an omnidirectionally stretchable ZAB was designed by engineering each individual battery component to be stretchable. Notably, a tough guanosine‐based hydrogel with high stretchability and conductivity was used as both the electrolyte and electrode binders to fabricate the stretchable ZAB, enabling robust conductivity interfaces between the different components of the battery even during extensive mechanical deformation processes. As a result, the obtained ZAB exhibited superior omnidirectional stretchability with an aerial strain of over 1000% at the full‐cell level. Moreover, the ZAB displayed excellent mechanical and electrochemical stability, withstanding harsh mechanical and environmental challenges such as undergoing more than 10 000 stretching cycles. Even at ultra‐low temperatures (down to −60 °C) and under continuous water immersion (>3 h) or rinsing (>5 h), the ZAB still performed well without any pre‐treatment. This study presents a combination of strategies involving active material design and device engineering to develop a high‐performance, omnidirectionally stretchable, waterproof, and low temperature‐tolerant device platform for future on‐skin wearable applications.

## 2 Results and Discussion

**Figure 1:**
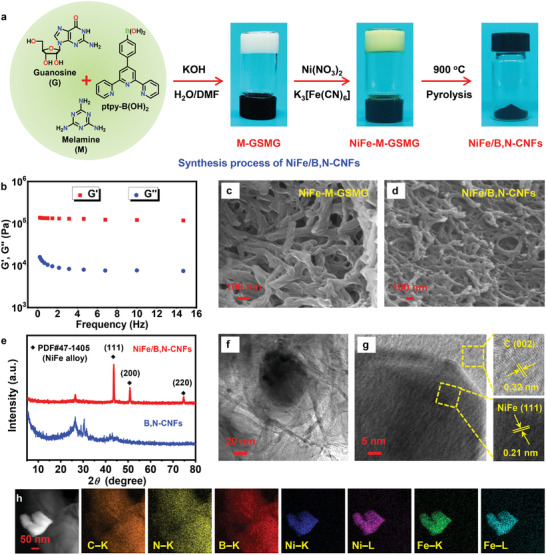
a) Synthesis process of NiFe/B,N‐CNFs. b) Rheological test of the supramolecular gel (2% w/v M‐GMSM). c,d) SEM images of NiFe‐M‐GSMG and NiFe/B,N‐CNFs. e) XRD patterns of NiFe/B,N‐CNFs and B,N‐CNFs. f,g) TEM and HRTEM images of NiFe/B,N‐CNFs. h) HAADF–STEM image and the corresponding EDX maps of NiFe/B,N‐CNFs.

Scheme 1 presents the design strategy for fabricating the omnidirectional stretchable ZAB, beginning with the design and synthesis of a highly efficient NiFe/B,N‐CNFs bifunctional electrocatalyst. The catalyst was obtained via the facile self‐templated conversion of a pre‐prepared GSMG developed in our previous work.^[^ ^31^ ^]^ The detailed synthetic procedure is shown in Supporting Information. Additional melamine was induced as a nitrogen source into GSMG, which was composed of guanosine and 4ʹ‐(4‐boronatophenyl)‐2,2ʹ:6ʹ,2ʺ‐terpyridine (ptpy‐B(OH)_2_, 1:1 molar ratio) in KOH DMF–H_2_O (1:1 v/v) solution (**Figure** **1**a). The obtained melamine‐doped GSMG (M‐GSMG) showed a typical fibrous structure of supramolecular gels (Figure S1a, Supporting Information). Rheological tests showed that the storage modulus (G’) of M‐GSMG was always larger than its loss modulus (G’’) over a large frequency and strain range, demonstrating its gel characteristics (Figure 1b and Figure S2, Supporting Information). Subsequently, Ni^2+^ and [Fe(CN)_6_]^3−^ ions were introduced to coordinate with the terpyridine groups on the fiber surface to further enhance the structural stability of M‐GSMG (Figure 1a). The resulting Ni^2+^ and [Fe(CN)_6_]^3−^‐doped M‐GSMG (NiFe‐M‐GSMG) maintained the original fibrous morphology of M‐GSMG, with an average diameter of ca. 60 nm (Figure 1c). After pyrolysis at 900 °C, the resulting NiFe/B,N‐CNFs inherited the continuous network of fibers of the precursor NiFe‐M‐GSMG (Figure 1d). By contrast, B,N‐CNFs derived from the metal‐free M‐GSMG displayed a fracture fiber morphology (Figure S1b, Supporting Information). The powder X‐ray diffraction (XRD) patterns of B,N‐CNFs exhibited a relatively broad diffraction peak at about 26.5°, attributed to graphitic carbon. In addition, melamine in the precursor material provides a rich nitrogen source, so the XRD pattern shows the diffraction peaks of carbon and nitrogen compounds species at about 30° (Figure 1e).^[^ ^32^ ^]^ The XRD pattern of NiFe/B,N‐CNFs displayed additional sharp diffraction peaks located at 43.7°, 50.8°, and 74.8°, which respectively corresponded to the (111), (200), and (220) lattice planes of NiFe alloy (JCPDS no. 47–1405) (Figure 1e) and confirmed the formation of the NiFe alloy during the pyrolysis process. Control experiments showed that the metal precursors were only able to be completely converted into NiFe alloy when the calcination temperature was higher than 800 °C (Figure S3, Supporting Information). Transmission electron microscope (TEM) images of NiFe/B,N‐CNFs (Figure 1f and Figure S4, Supporting Information) clearly showed that the NiFe alloy nanoparticles were uniformly dispersed and embedded in situ inside the porous carbon matrix formed by the carbon fiber network. Such a geometric structure would confine the active particles within an interconnected framework, and possibly stabilize the NiFe active component with a large surface area.^[^ ^33^ ^]^ High‐resolution TEM (HRTEM) images (Figure 1g) confirmed that the NiFe alloy nanoparticles had a *d*‐spacing of 0.21 nm, corresponding to the (111) crystal planes of the NiFe alloy.^[^ ^34^ ^]^ The NiFe alloy nanoparticles were encapsulated by a few‐layer graphene carbon shell with a layer spacing of 0.32 nm, which was attributed to the (002) plane of graphene carbon.^[^ ^35^ ^]^ Such an intimate contact can not only inhibit the agglomeration and dissolution of metal particles during continuous electrochemical cycling but also effectively accelerate the electron transfer during electrocatalytic processes.^[^ ^36^, ^37^ ^]^ A high‐angle annular dark‐field scanning transmission electron microscopy (HAADF–STEM) image and the corresponding energy‐dispersive X‐ray spectroscopy (EDX) maps (Figure 1h) verified the uniform distribution of N and B in the carbon nanofibers and Ni and Fe in the alloy phase.

**Figure 2:**
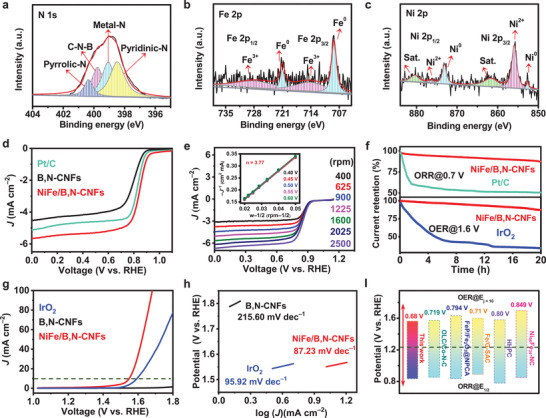
a–c) High‐resolution N 1s (a), Fe 2p (b), and Ni 2p (c) XPS spectra of NiFe/B,N‐CNFs. d) ORR polarization curves of NiFe/B,N‐CNFs, B,N‐CNFs, and Pt/C. e) LSV curves of NiFe/B,N‐CNFs at different rotation speeds. Inset: K‐L plots. f) Stability profiles of NiFe/B,N‐CNFs for ORR and OER in comparison with Pt/C and IrO_2_. g) OER polarization curves of NiFe/B,N‐CNFs, B,N‐CNFs, and IrO_2_. h) Tafel curves of NiFe/B,N‐CNFs, B,N‐CNFs, and IrO_2._ i) Potential differences between the E_1/2_ of ORR and E_j = 10_ of OER.^[^ ^24^, ^25^, ^45^, ^46^, ^47^ ^]^

The surface composition and chemical binding states of NiFe/B,N‐CNFs were analyzed by X‐ray photoelectron spectroscopy (XPS). The typical C 1s, N 1s, B 1s, Fe 2p, and Ni 2p peaks were clearly observed in the XPS survey spectrum (Figure S5, Supporting Information). The high‐resolution C 1s XPS spectrum (Figure S6, Supporting Information) was indexed into four peaks representing C—C (284.5 eV), C—B (285.2 eV), C—N (286.0 eV), and C═O (287.8 eV) bonds.^[^ ^38^ ^]^ The N 1s spectrum is shown in **Figure** **2**a, and the peaks located at 398.5 and 400.4 eV were assigned to pyridinic and pyrrolic N, respectively.^[^ ^39^ ^]^ The other two peaks located at 399.1 and 399.8 eV were attributed to metal–N and C—N—B, respectively.^[^ ^31^, ^40^ ^]^ Correspondingly, C—B (190.1 eV) and N—B (190.72) peaks were also displayed in the high‐resolution B 1s spectrum (Figure S7, Supporting Information). These results revealed the successful doping of N and B into the carbon matrix of NiFe/B,N‐CNFs, which provided additional active sites for the prepared materials. Furthermore, the Fe 2p spectrum was deconvoluted into four peaks. These were Fe^3+^ peaks at 714.1 (Fe 2p_3/2_) and 727.7 eV (Fe 2p_1/2_) as well as metallic iron peaks at 708.3 eV (Fe 2p_3/2_) and 721.0 eV (Fe 2p_1/2_) (Figure 2b). The Ni 2p spectrum (Figure 2c) showed two peaks at 855.9 and 876.4 eV that were attributed to the Ni 2p_3/2_ and Ni 2p_1/2_ signals of Ni^2+^ and two other peaks at 852.6 and 873.1 eV that were assigned to metallic Ni species. The remaining pair of weak peaks located at 861.7 and 880.4 eV were satellite peaks.^[^ ^33^ ^]^ These results confirmed the coexistence of the bimetallic NiFe alloy with the B,N‐doped graphitic carbon in NiFe/B,N‐CNFs. The presence of high valence states of Fe and Ni may be due to the formation of metal‐N bonds or surface oxidation.^[^ ^33^, ^41^ ^]^ It was worth noting that the signals in the XPS spectra of Fe 2p and Ni 2p were weak, which might be because the encapsulated carbon layers hindered the detection of NiFe by XPS. In addition, heteroatom substitution, vacancies, and grain boundaries are generally considered to be important factors that induce defects.^[^ ^42^ ^]^ After the introduction of NiFe, the D/G band ratio of B,N‐CNFs increased from 1.01 to 1.04, suggesting a more defective structure (Figure S8, Supporting Information),^[^ ^43^ ^]^ which is beneficial for the formation of more potential active sites for electrocatalysis. The N_2_ adsorption–desorption analyses of the NiFe/B,N‐CNFs featured a type IV isotherm with clear hysteresis (Figure S9, Supporting Information), implying the existence of mesopores. The average pore size was determined to be ca. 4.0 nm (Figure S10, Supporting Information), and the Brunauer–Emmett–Teller specific surface area was calculated to be 125 m^2^ g^−1^. This highly porous structure combined with rich mesopores was expected to be beneficial for active site exposure and mass transfer during electrochemical processes,^[^ ^44^ ^]^ demonstrating the high potential of NiFe/B,N‐CNFs for use as an electrocatalyst.

As a proof of concept, the ORR and OER performance of NiFe/B,N‐CNFs was evaluated in O_2_‐saturated KOH solution (Figure 2). In the ORR tests, cyclic voltammetry (CV) and linear sweep voltammetry (LSV) measurements revealed that NiFe/B,N‐CNFs displayed superior ORR activity, with a high onset potential (*E*_onset_) and a high half‐wave potential (*E*_1/2_) of about 0.94 and 0.84 V, respectively (Figure 2d and Figure S11, Supporting Information). Both of these values are higher than those of the metal‐free B,N‐CNFs (*E*_onset_ = 0.89; *E*_1/2_ = 0.77 V) and a commercial Pt/C catalyst (*E*_onset_ = 0.91; *E*_1/2_ = 0.82 V). Moreover, NiFe/B,N‐CNFs exhibited the lowest Tafel slope (78.56 mV dec^−1^) compared to B,N‐CNFs (115.65 mV dec^−1^) and Pt/C (82.35 mV dec^−1^), suggesting favorable ORR kinetics (Figure S12, Supporting Information). The Koutecky–Levich (K‐L) plot obtained from the LSV curves measured at different rotation speeds (400 to 2500 rpm) revealed that the electron transfer number (n) of each O_2_ molecule was about 4 (Figure 2e), implying that NiFe/B,N‐CNFs was able to catalyze the ORR through direct reduction.^[^ ^48^ ^]^ In addition, NiFe/B,N‐CNFs delivered a much lower overpotential (*E*_j = 10_) of approximately 1.52 V at a current density of 10 mA cm^−2^ compared to that of an IrO_2_ catalyst (1.56 V) and the metal‐free B,N‐CNFs (>1.8 V) (Figure 2g), suggesting superior OER electrocatalytic activity. The relatively low charge transfer resistance (*R*_ct_) and OER Tafel slope of NiFe/B,N‐CNFs (19.87 Ω, 87.23 mV dec^−1^) compared with those of B,N‐CNFs (44.95 Ω, 215.60 mV dec^−1^) and IrO_2_ (27.86 Ω, 95.92 mV dec^−1^) suggest that NiFe/B,N‐CNFs has a faster charge transfer ability and accelerated OER kinetics (Figure 2h and Figure S13, Table S1, Supporting Information). The relatively high double‐layer capacitance (*C*_dl_) value of NiFe/B,N‐CNFs (5.15 mF cm^−2^) calculated from the CV curves in the non‐faradaic region compared to those of B,N‐CNFs (0.26 mF cm^−2^) and IrO_2_ (2.10 mF cm^−2^) further prove that NiFe/B,N‐CNFs has a large electrochemical surface area (ECSA) with more exposed catalytic active sites. This is responsible for its superior catalytic activity (Figure S14, Supporting Information). As a reversible O_2_ electrocatalyst, NiFe/B,N‐CNFs exhibited a much low ORR/OER potential gap (Δ*E* = *E*_j = 10_ − *E*_1/2_) of only 0.68 V, significantly outperforming the noble‐metal‐based Pt/C + IrO_2_ benchmark (Δ*E* = 0.74 V) and most other reported bifunctional electrocatalysts (Figure 2i and Table S2, Supporting Information). In particular, NiFe/B,N‐CNFs also exhibited superior durability for both ORR and OER. The relative current retention of NiFe/B,N‐CNFs after 20 h was 87.5% for the ORR and 87.2% for the OER, far surpassing that of benchmark Pt/C and IrO_2_ catalysts, respectively (Figure 2f). Based on the above results, we can conclude that NiFe/B,N‐CNFs provide excellent bifunctional performances for both ORR and OER, which may be mainly attributed to the multi‐synergistic effects between metallic NiFe, porous carbon nanofiber structure, and abundant B,N‐doping. The well‐dispersed NiFe nanoparticles and abundant N‐doping in the NiFe/B,N‐CNFs provide more accessible active sites for both ORR/OER.^[^ ^49^ ^]^ The interconnected porous architecture offers abundant transport channels for fast reactants permeation and diffusion, large surface area for more catalytically active sites, and efficient charge transfer during the ORR/OER.^[^ ^50^ ^]^ The B‐doping makes the surrounding metal centers less positive and weakens the interaction between the metal centers and the adsorbed intermediates which improves the reaction dynamics and increases the reaction efficiency.^[^ ^50^ ^]^ Besides, the encapsulated carbon matrix outside the NiFe alloy nanoparticles also effectively suppresses particle agglomeration and dissolution during continuous electrochemical cycling, leading to long‐term stability. The ultrahigh bifunctional catalytic activity and remarkable stability of the rationally designed NiFe/B,N‐CNFs are expected to ensure good electrochemical performance for ZABs under extreme working conditions.^[^ ^51^ ^]^

**Figure 3:**
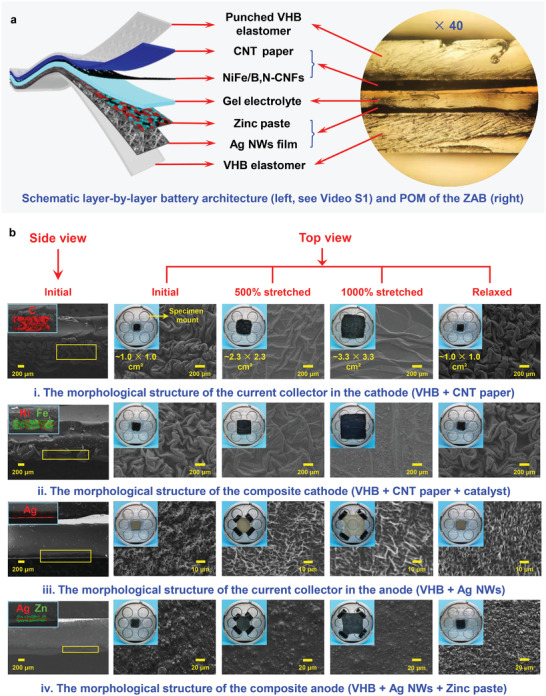
a) The layer‐by‐layer architecture and POM of the ZAB (see Video S1, Supporting Information). b) SEM images of each component of the ZAB without or with varying mechanical strain (stretched to 500% or 1000%). Inset: the corresponding EDX images and experimental site photographs.

Based on the concept design proposed in Scheme 1, an omnidirectionally stretchable ZAB was assembled. The structure and fabrication of the stretchable ZAB are schematically shown in **Figure** **3**a and Video S1, Supporting Information. Considering the desirable comprehensive properties of high elasticity, toughness, and superior interface adhesiveness, we chose a stretchable acrylic elastomer (3 m VHB 4910, double‐sided adhesive transparent, 1.0 mm thick) as the flexible substrate in this work. For the preparation of the stretchable air cathode, the guanosine supramolecular gel‐derived NiFe/B,N‐CNFs bifunctional catalyst was coated onto the carbon nanotube (CNT) paper and directly attached to the center of the biaxially pre‐strained VHB tape, causing crumpling upon strain release and producing a stretchable electrode with superior isotropic stretchability (Figure 3b). The VHB elastomer was punched for efficient gas diffusion. To prepare the anode, a spray coating method was first developed to deposit Ag nanowires (NWs) with a thickness of ca. 30 µm and a length of several micrometers on the biaxially pre‐strained VHB substrate to fabricate a stretchable current collector (Figure 3b and Figure S15, Supporting Information). The sheet resistance of the VHB + Ag NWs substrate was measured to be only 0.93 at 0% areal strain and 4.07 Ω sq^−1^ at 1000% areal strain (Table S3, Supporting Information), ensuring efficient electronic transmission. Then, an anode paste composed of zinc powder, carbon black, and a small amount of guanosine‐based hydrogel as a binder was tightly bound to the VHB + Ag NWs substrate through a slurry‐casting method, resulting in a high mass loading of 60 mg cm^−2^ and forming an intrinsically stretchable composite electrode with an aerial strain capability of over 1000% (Figure 3b). Notably, the binder for the active materials coated on the VHB + Ag NWs current collectors was a highly stretchable and ionically conductive guanosine‐based supramolecular‐polymer double‐network (SP‐DN) hydrogel with superior impressive interface adhesiveness and an ultra‐low temperature tolerance (−196 °C, liquid nitrogen), as reported in our previous work.^[^ ^13^ ^]^ Due to its excellent strain accommodation abilities, this elastic SP‐DN hydrogel binder offered additional advantages in terms of improving the strain performance of electrode materials in stretchable batteries compared with the more commonly used stiff and insulating polymeric binders such as PVDF.^[^ ^7^, ^52^, ^53^, ^54^ ^]^ Additionally, this highly stretchable and conductive SP‐DN hydrogel was also employed as an intrinsically stretchable semisolid electrolyte to separate the cathode and anode (Figure 3a). As a result, each individual component of the ZAB was stretchable, allowing for the assembly and fabrication of a highly stretchable full cell. This innovative cell design enabled by the use of the guanosine‐based hydrogel in both the electrode and electrolyte ensured the creation of a highly stretchable ZAB with robust conductive interfaces able to withstand extensive mechanical stress while maintaining efficient ion and electron transport properties in both the lateral and transversal directions. Polarized optical micrographs (POM) clearly displayed the layer‐by‐layer battery architecture and the close contact of each battery component (Figure 3a), which was further confirmed by cross‐sectional scanning electron microscopy (SEM) images and EDX mapping (Figure 3b). In detail, for the cathode, wrinkled CNT paper was obtained in the initial state. When the tensile area strain reached 500% and 1000%, the CNT paper gradually spread out, and no appreciable cracks were observed (Figure 3b‐i and ‐ii). For the anode, the coated Ag NWs also formed crumpled film after releasing the pre‐strained VHB elastomer substrate. After stretching to areal strains of 500% and 1000%, the Ag NWs were still continuous and interwoven, ensuring good conductivity under stretching conditions (Figure 3b‐iii). With further coating of the Zn anode paste, obviously agglomerated particles were observed in the initial state, which gradually became uniformly distributed during the continuous stretching process (Figure 3b‐iv). Furthermore, no delamination of the active materials from the substrate was visible for both the cathode and anode, confirming the robust and well‐adhered interfaces between the different components of the ZAB (Figure 3b). After releasing the mechanical stress, the relaxed current collectors and electrodes exhibited similar morphology compared with that of the initial state, indicating excellent reversible capability (Figure 3b).

**Figure 4:**
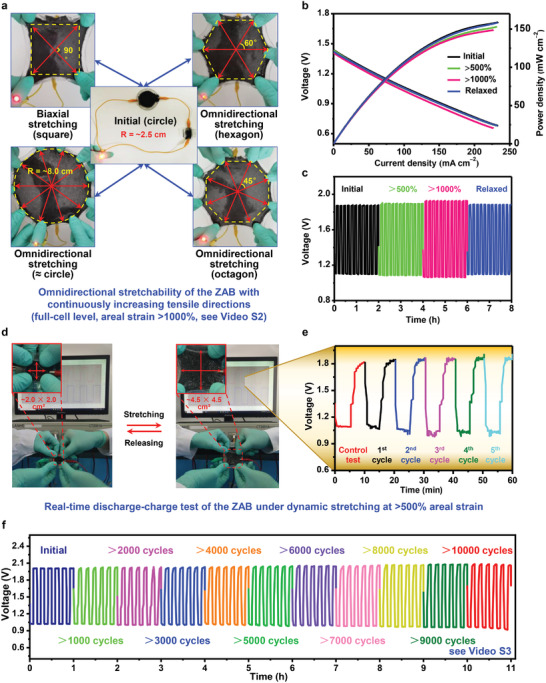
a) Demonstration of the omnidirectional stretchability of the ZAB (see Video S2, Supporting Information). b) Discharge and power density curves of the ZAB at various areal strains. c) Cycling profiles of the ZAB at various areal strains with a current density of 2.0 mA cm^−2^. d,e) Experimental site photographs and real‐time discharge and charge test curve of the ZAB during a repeated dynamic stretching‐releasing process at room temperature. f) Cycling stability of the ZAB during 10 000 stretching cycles at an aerial strain of ca. 400% (see Video S3, Supporting Information).

Benefiting from the innovative cell design, the fabricated ZAB exhibited excellent ductility and was easily stretched omnidirectionally (**Figure** **4**a and Video S2, Supporting Information). Notably, a circular ZAB with a radius of ≈2.5 cm was easily stretched to ≈8.0 cm under omnidirectional tension, achieving an aerial strain of over 1000% at the full‐cell level (Figure 4a and Video S2, Supporting Information). Without mechanical strain, the stretchable ZAB showed excellent electrochemical performance that outperformed that of many aqueous regular ZABs at room temperature.^[^ ^55^, ^56^, ^57^, ^58^ ^]^ The ZAB delivered a stable open‐circuit voltage of ≈1.47 V (Figure S16, Supporting Information), a high power density of 159.0 mW cm^−2^ (Figure 4b), extraordinary cycling stability (> 130 h), and excellent rate discharge properties (Figures S17 and S18, Supporting Information). Upon stretching to areal strains of 500% and 1000%, the ZAB still exhibited comparable power densities of 153.0 and 148.8 mW cm^−2^, respectively (Figure 4b and Figure S19, Supporting Information). Moreover, the corresponding rechargeable characteristics of the stretched ZAB were well‐maintained with only subtle changes (Figure 4c). The ZAB battery also showed excellent resilience. Once the stress was released, both the configuration and the electrochemical performance of the stretched ZAB were immediately restored to their initial states, showing great promise for practical application (Figure 4b,c, Figure S19, and Video S2, Supporting Information). More importantly, the electrochemical performance of the ZAB was also monitored in situ under dynamic stretching‐releasing modes to simulate real situations for practical wearable applications. As shown in Figure 4d,e, the ZAB was capable of stably charging and discharging within the voltage range of 1.00–1.86 V at 2 mA cm^−2^ while being subject to dynamic stretching and releasing at an aerial strain of >500%, suggesting that it could reliably output power upon deformation. As a result, the ZAB was able to continuously power a red LED during the omnidirectional stretching‐releasing process (Video S2, Supporting Information). Furthermore, the assembled ZAB withstood more than 10 000 stretching–releasing cycles at a ≈400% areal strain while maintaining stable rechargeability (Figure 4f and Video S3, Supporting Information). This far exceeds the number of mechanical loading cycles reported in previous works,^[^ ^59^, ^60^ ^]^ indicating the excellent electrochemical stability and mechanical durability of our ZAB upon strain cycling.

**Figure 5:**
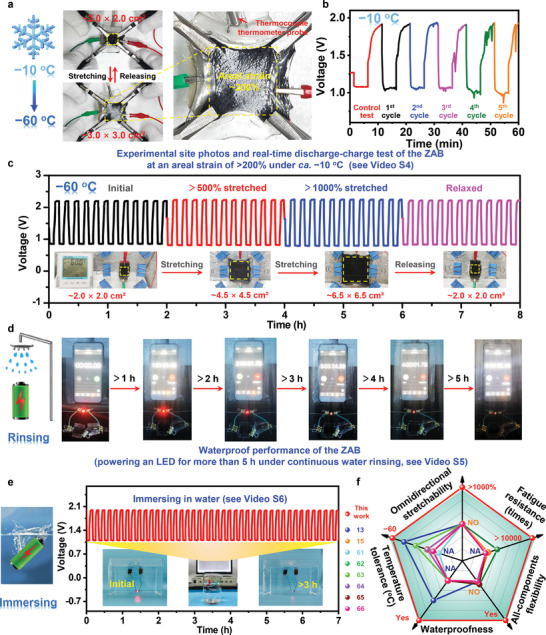
a,b) Experimental site photographs and corresponding real‐time discharge and charge test curves of the ZAB during repeated dynamic stretching‐releasing process at −10 °C (see Video S4, Supporting Information). c) Galvanostatic discharge and charge profiles of the ZAB under the initial, 500% stretched, 1000% stretched, and relaxed configurations at −60 °C. Inset: experimental site photographs. d) Waterproof performance test of the ZAB, which powered an LED for more than 5 h under continuous rinsing (see Video S5, Supporting Information). e) In situ galvanostatic cycling profiles of the ZAB at 2 mA cm^−2^ while immersed in water. Inset: experimental site photographs of the ZAB for the waterproof performance test. The ZAB continuously powered an LED for more than 3 h while immersed in water (see Video S6, Supporting Information). f) Comparison of the omnidirectionally stretchable ZAB with previously reported batteries regarding stretchability, fatigue resistance, flexible strategy, waterproofness, and ultra‐low temperature tolerance.

More importantly, the ZAB also exhibited outstanding mechanical and electrochemical stability to withstand extreme environmental conditions. At an ultra‐low temperature down to −20 °C, the ZAB (in thermal equilibrium state) still provided remarkable electrochemical performance comparable with that of its room‐temperature counterpart (Figures S20–S22, Supporting Information). Moreover, the ZAB still retained superior reversible stretchability with an aerial strain of >200% at an environment temperature of ca. −20 °C (**Figure** **5**a and Video S4, Supporting Information). It was worth noting that the real‐time temperature of the ZAB during stretching process was about −10 °C. In situ discharge‐charge test indicated that the electrochemical cycling performance of the ZAB remained unaffected for at least 5 consecutive dynamic stretching‐releasing cycles at around −10 °C (Figure 5a,b and Video S4, Supporting Information). Unfortunately, limited by the operating temperature of the VHB elastomer used to construct the ZAB, the battery was more difficult to freely stretch at lower temperatures. However, the ZAB still maintained its highly efficient electrochemical performance and stable rechargeability characteristics under 0%, 500%, and 1000% stretched configurations, even down to −60 °C (Figure 5c). The obtained power density of the ZAB at −60 °C was as high as 94.2, 91.2, and 88.5 mW cm^−2^ under areal strains of 0%, 500%, and 1000%, respectively (Figures S23 and S24, Supporting Information). A galvanostatic discharge‐charge test further showed that the ZAB initially generated a narrow voltage gap of 0.78 V and that after 510 cycles, no obvious voltage fading was visible, demonstrating its superior long‐term durability in harsh conditions (Figure S25, Supporting Information). More inspiringly, the ZAB, without any additional packaging or treatment, was able to power an LED for more than 5 h under continuous rinsing with tap water (Figure 5d and Video S5, Supporting Information). The ZAB also continuously operated for more than 3 h while immersed in water (Video S6, Supporting Information), demonstrating outstanding waterproof performance. An in situ electrochemical test demonstrated that after 3 h of immersion in water, the ZAB still delivered a comparable power density relative to the initial ZAB (Figures S26 and S27, Supporting Information). An in situ discharge‐charge test further indicated that the ZAB displayed unprecedented cycling capability while immersed in water, maintaining a voltage gap of 0.88 V for 42 cycles (>7 h at 2 mA cm^−2^) (Figure 5e). These results clearly demonstrate the superior environmental stability of the stretchable ZAB under both ultra‐low temperature and underwater conditions. A comparison with previously reported flexible batteries is provided in Figure 5f and Table S4, Supporting Information.^[^ ^13^, ^15^, ^61^, ^62^, ^63^, ^64^, ^65^, ^66^ ^]^ To the best of our knowledge, this ZAB is the first flexible battery that exhibits omnidirectional stretchability with a high areal strain >1000% as well as superb mechanical and electrochemical stability, with the ability to withstand ultra‐low temperatures and continuous water rinsing or immersion.

## 3 Conclusion

In summary, we have successfully developed an omnidirectionally stretchable ZAB with excellent electrochemical performance and superior mechanical and environmental adaptability through the synergistic engineering of the battery's active materials and device architecture. A highly efficient bifunctional NiFe/B,N‐CNFs ORR/OER electrocatalyst was first designed and synthesized via the self‐templated conversion of a GSMG to ensure the excellent electrochemical performance of the ZAB even under harsh mechanical and environmental conditions. An innovative cell design that used a highly stretchable and conductive guanosine‐based hydrogel in both the electrode and the electrolyte allowed for the fabrication of a highly stretchable ZAB with robust conductive interfaces that could withstand extensive mechanical stress. This combined strategy endowed the assembled ZAB with omnidirectional stretchability under a high areal strain of >1000% at the full‐cell level. The ZAB maintained stable rechargeability even after undergoing more than 10 000 dynamic stretching cycles More interestingly, even at ultra‐low temperatures (down to −60 °C) and under continuous water immersion (>3 h) or rinsing (>5 h), the highly efficient and stable electrochemical performance of the ZAB was still well‐preserved. The presented strategy would greatly benefit the design and fabrication of high‐performance omnidirectional stretchable batteries with extreme environmental adaptability for the next generation of wearable soft electronics.

## 4 Experimental Section

### Synthesis of NiFe‐M‐GSMG

NiFe‐M‐GSMG was synthesized by a slightly modified method reported in the previous work.^[^ ^31^ ^]^ In detail, 24.7 mg 4ʹ‐(4‐boronatophenyl)‐2,2ʹ:6ʹ,2ʺ‐terpyridine (ptpy‐B(OH)_2_, 0.07 mmol) and 20.0 mg melamine were added to a clean reaction cell followed by the addition of 1.0 mL 0.07 m KOH DMF–H_2_O (volume ratio 1:1) solution. The resulting suspension was heated to obtain a homogenous solution. Next, 20.0 mg guanosine (G, 0.07 mmol) was instantly added to the vial and dissolved by gentle heating to obtain a clear solution. After naturally cooling the solution to room temperature, an opaque melamine‐doped guanosine‐based supramolecular hydrogel (M‐GSMG) (2% w/v in G) was formed within 5 min. After forming the hydrogel, 0.5 mL K_3_Fe[(CN)_6_]–Ni(NO_3_)_2_ 6H_2_O (0.28 m, molar ratio 1:1) solution in DMF–H_2_O (volume ratio 1:1) mixture solvent was added to the top of the as‐prepared M‐GSMG sample to allow for natural penetration. After 12 h, yellow NiFe‐M‐GSMG samples were obtained. As a control, metal‐free M‐GSMG samples were also prepared by following a similar procedure without adding K_3_Fe[(CN)_6_] and Ni(NO_3_)_2_ 6H_2_O.

### Synthesis of NiFe/B,N‐CNFs Catalyst

The NiFe/B,N‐CNFs catalyst was synthesized by pyrolyzing the NiFe‐M‐GSMG samples in a tube furnace under an Ar atmosphere at 900 °C for 2 h with a ramp rate of 5 °C min^−1^. As a control, metal‐free B,N‐CNFs were also synthesized via the self‐templated conversion of M‐GSMG samples under the same conditions. Other calcination temperatures were also investigated to optimize the synthesis conditions.

### Fabrication of the Omnidirectionally Stretchable ZAB

First, 10.0 mg NiFe/B,N‐CNFs catalyst was dispersed in a mixed solution containing 580 µL H_2_O, 380 µL ethanol, and 40 µL Nafion (5.0 wt%) under sonication for 1 h to form a homogeneous catalyst ink. The ink was then dripped onto a CNT paper (≈7.0 cm × 7.0 cm). The mass loading of the catalyst on the CNT paper was ca. 0.24 mg cm^−2^. After drying, the catalyst‐loaded CNT paper was attached to an omnidirectionally pre‐stretched and punched VHB elastomer (>1000% areal strain) and served as the air cathode upon strain release. Next, 1.0 mL Ag NWs (50 mm in diameter) stock solution was dispersed in 9.0 mL ethanol by sonication for 0.5 h to ensure uniform mixing. The obtained Ag NWs solution was spray‐coated onto the biaxially pre‐stretched VHB elastomer (>1000% areal strain) by using an airbrush gun (nozzle diameter: 0.35 mm) to form a stretchable VHB + Ag NWs current collector after strain release. Next, anode paste composed of zinc powder, carbon black, and a small amount of guanosine‐based supramolecular‐polymer double‐network (SP‐DN) hydrogel (reported by the previous work)^[^ ^13^ ^]^ as a binder (mass ratio of 40:1:20) was prepared by grinding, then tightly bound to the VHB + Ag NWs substrate through a slurry‐casting method (coating area = ≈2.0 cm × 2.0 cm, mass loading of 60 mg cm^−2^) to form an intrinsically stretchable composite electrode. This highly stretchable conductive SP‐DN hydrogel was also employed as an intrinsically stretchable semisolid electrolyte to separate the cathode and anode in the ZAB. Consequently, each individual component of the ZAB was stretchable, and a highly stretchable full cell with omnidirectional stretchability was assembled. Following this method, a ZAB without mechanical strain was obtained with a size of ca. ≈2.0 cm × 2.0 cm. This ZAB could be stretched to ≈7.0 cm × 7.0 cm with an areal strain of over 1000%. Other ZAB sizes and shapes could be prepared following a similar procedure.

## Conflict of Interest

The authors declare no conflict of interest.

## Supporting information

Supporting Information

Supporting Video 1

Supporting Video 2

Supporting Video 3

Supporting Video 4

Supporting Video 5

Supporting Video 6

## Data Availability

The data that support the findings of this study are available from the corresponding author upon reasonable request.
