# Supplementary material for: Supramolecular Gel‐Derived Highly Efficient Bifunctional Catalysts for Omnidirectionally Stretchable Zn–Air Batteries with Extreme Environmental Adaptability
Source: Adv Sci (Weinh). 2022 May 6;9(20):2200753. doi: 10.1002/advs.202200753 (PMC9284165; doi:10.1002/advs.202200753)
Supplement: Supplementary file 1 — Supporting Information [file ADVS-9-2200753-s003.pdf]

ADVANCED SCIENCE
Open Access

Supporting Information

for *Adv. Sci.*, DOI 10.1002/advs.202200753

Supramolecular Gel-Derived Highly Efficient Bifunctional Catalysts for Omnidirectionally Stretchable Zn–Air Batteries with Extreme Environmental Adaptability

Junpeng Liu, Mengke Wang, Chaonan Gu, Jingjing Li, Yujia Liang, Hai Wang, Yihan Cui and Chun-Sen Liu**

Supporting Information

Supramolecular Gel-Derived Highly Efficient Bifunctional Catalysts for Omnidirectionally Stretchable Zn–Air Batteries with Extreme Environmental Adaptability

Junpeng Liu[1+], *Mengke Wang*[1+], *Chaonan Gu*,[1] *Jingjing Li*[2,]*, *Yujia Liang*[1], *Hai Wang*[1], *Yihan Cui*[1], *and Chun-Sen Liu*[1,]*

[1] Henan Provincial Key Lab of Surface & Interface Science, Zhengzhou University of Light Industry, Zhengzhou, 450001, China.

[2] School of Chemistry and Chemical Engineering, Henan University of Technology, Zhengzhou, 450001, China.

[+] These authors contributed equally to this work.

* ✉ Correspondence to: nicoleljj@tju.edu.cn (J. Li); nkchunsenliu@163.com (C.-S. Liu)

Table of Contents

Section S1 Methods

Section S2 Structural characterization and catalytic activity of NiFe/B,N-CNFs

Section S3 Electrochemical performance of the omnidirectionally stretchable ZAB at room temperature.

Section S4 Environmental adaptability of the omnidirectionally stretchable ZAB.

Section S5 References

Video S1

The manufacturing process of the omnidirectionally stretchable ZAB.

Video S2

Demonstration of the omnidirectional stretchability of the ZAB with continuously increasing tension directions (full-cell level, areal strain >1000%).

Video S3

Anti-fatigue performance of the ZAB, which can withstand more than 10000 stretching-releasing cycles while maintaining stable rechargability.

Video S4

Real-time discharge and charge test of the ZAB under dynamic stretching at −10 °C.

Video S5

Waterproof performance of the ZAB without any special treatment, which can power a light-emitting diode (LED) for more than 5 h under continuous water rinsing.

Video S6

Waterproof performance of the ZAB without any special treatment, which can continuously power an LED for more than 3 h while immersed in water.

Section S1 Methods

1. Chemicals and Materials.

Guanosine (G, 98%) was purchased from Alfa aesar. Melamine was purchased from Kermel. Nickel nitrate hexahydrate ($Ni(NO_3)_2 \cdot 6H_2O$, 98%) and Potassium ferricyanide ($K_3[Fe(CN)_6]$, ⩾99%) were purchased from Aladdin. Commercial 20 wt% Pt/C and IrO_2 were purchased from Sigma-Aldrich. KOH (99.98%) was purchased from Acros Organics Co., Ltd.. Carbon nanotube (CNT) paper were purchased from Suzhou Tanfeng Graphene Technology Co., Ltd.. Zinc powder (purity > 99.99%) were purchased from Sinopharm Chemical Reagent Co. Ltd. Ag nanowires (Ag NWs, 50 nm in diameter) were purchased from XFnano Co. Ltd. All other chemicals used in this work were commercially analytical grade reagents without further purification.

2. Instruments

The morphological structures of the samples were characterized by scanning electron microscopy (SEM, JEOL, JSM-6490LV). Transmission electron microscopy (TEM), high resolution transmission electron microscopy (HRTEM) and high-angle annular dark-field scanning transmission electron microscopy (HAADF–STEM) were captured on the JEOL, JEM-2100 instrument at an acceleration voltage of 200 kV. X-ray diffraction (XRD) patterns were recorded on a Rigaku D/Max-2500 X-ray diffractometer with Cu Kα radiation. Raman spectra were obtained on a Renishaw *via* Raman spectrometer with a solid-state laser (λ = 532 nm). The X-ray photoelectron spectroscopy (XPS) experiments were conducted on an ESCALAB 250Xi spectrometer. The Brunauer-Emmett-Teller (BET) specific surface areas were calculated from nitrogen adsorption/desorption isotherms under ultrahigh vacuum at 77 K using a Micromeritics 3Flex surface area and pore size analyzer. The sheet resistance was determined by the four-point probe method (Guangzhou Four-Probe Technology, RTS-9).

3. Electrochemical characterizations

All electrochemical measurements were performed on a ModuLab XM electrochemical workstation with a standard three electrodes system at room temperature. A rotating disk electrode (RDE) with 5 mm in diameter loaded with different catalysts was used as working electrode, Pt wire was used as the counter electrode, and a saturated Ag/AgCl electrode was served as the reference electrode. All measured potentials in this work were converted to a reversible hydrogen electrode (RHE) scale, $E_{RHE} = E_{Ag/AgCl} + 0.059\ pH + E^{\theta}_{Ag/AgCl}$. For comparison, commercial Pt/C and IrO_2 catalysts were used as references for ORR and OER, respectively. For the preparation of the catalyst ink, 5.0 mg catalyst was ultrasonically dispersed for 1 h in a mixed solution containing of 360 μL DI water, 240 μL ethanol and 12 μL Nafion (5 wt%) solution to form a homogeneous catalyst ink. The loading mass was 0.42 mg cm^{-2} for all of the catalysts measured.

ORR measurements: All the measurements were conducted in the N_2 or O_2 saturated 0.1 M KOH electrolyte. The cyclic voltammetry (CV) measurements were carried out at a scan rate of 100 mv s^{-1}. Linear sweep voltammetry (LSV) tests were conducted at a scan rate of 10 mV s^{-1} with rotation speeds from 400 to 2500 rpm. Koutecky-Levich plots (J^{-1} *vs.* $\omega^{-1/2}$) were fitted into the linear curves, where the slopes and intercepts can be used to calculate the electron transfer number (n) and kinetic current density (J_k) number according to the following equations:

$$\frac{1}{J} = \frac{1}{J_L} + \frac{1}{J_K} = \frac{1}{B\omega^{\frac{1}{2}}} + \frac{1}{J_K} \qquad \text{-------- (1)}$$

$$B = 0.62nFC_0D_0^{2/3}\upsilon^{-1/6} \qquad \text{-------- (2)}$$

where J is the measured current density; J_L and J_k are the limiting and kinetic current densities, respectively; ω is the angular velocity; F is the Faraday constant (96486 C mol^{-1}); C_0 (1.2×10^{-6} mol cm^{-3})

and D_0 (1.9×10^{-5} cm^2 s^{-1}) are the bulk concentration and diffusion coefficient of O_2, respectively; v is the kinematic viscosity of the electrolyte (0.01 cm^2 s^{-1}). The stability of optimized catalyst and commercial catalyst were characterized by chronopotentiometric test at a fixed potential of 0.7 V (vs. RHE) and rotation speed of 1600 rpm in O_2 saturated electrolyte for 20 h.

OER measurements: All the measurements were conducted in 1.0 M KOH electrolyte. The LSV curves were measured at a potential window of 0–1 V (vs. RHE) with a sweep rate of 5 mV s^{-1} and all the LSV curves were presented with 90% iR compensation. The electrochemically active surface areas (ECSA) of the samples were roughly evaluated based on the electrochemical double-layer capacitance (C_{dl}), which was determined from CV curves recorded at different scan rates from 20 to 100 mV s^{-1}. The value of C_{dl} was calculated according to the following equation:

$$C_{dl} = \frac{J_a - J_c}{2v} = \frac{\Delta J}{2v} \qquad \text{-------- (3)}$$

where ΔJ is the difference between anodic and cathodic current densities recorded at the middle of selected potential range, and v is the scan rate. The electrochemical impedance spectroscopy (EIS) measurements were carried out at various overpotentials from 10 kHz to 0.1 Hz with an amplitude of 5 mV. The stability of optimized catalyst and commercial catalyst were characterized by chronopotentiometric test at a fixed potential of 1.6 V (vs. RHE) for 20 h.

4. Electrochemical measurements of the omnidirectionally stretchable ZAB

The open-circuit voltages, charge-discharge polarization and galvanostatic discharge curves of the ZAB were conducted using a Solartron Analytical Modulab XM CHAS 08 electrochemical workstation. The rate performance was determined at current densities of 1, 2, 4, 6, 8, and 10 mA cm^{-2}. The galvanostatic discharge and charge test was performed using a Land 2001A battery test system at a constant current density of 2 mA cm^{-2}, with each cycle consisting of 5 min for discharge followed by 5

min for charge.

For temperature-dependent electrochemical performance, the ZABs were tested at 25, −20, and −60 °C in a constant temperature chamber. The low temperatures of –20 and –60 °C were controlled *via* an ultra-low temperature Freezer (MELNG, DW-HW50). For waterproof performance, *in situ* electrochemical test were performed by completely immersing the ZAB in water.

Section S2 Structural characterization and catalytic activity of NiFe/B,N-CNFs

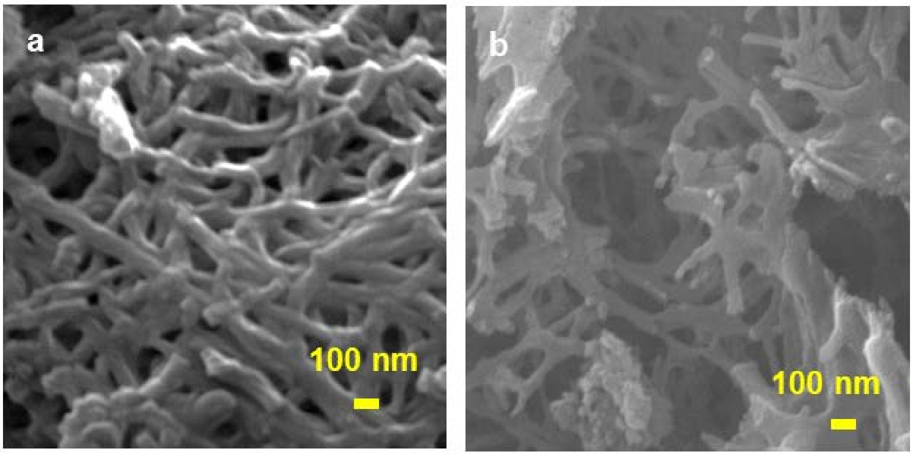

Figure S1. SEM images of (a) M-GMSG and (b) B,N-CNFs.

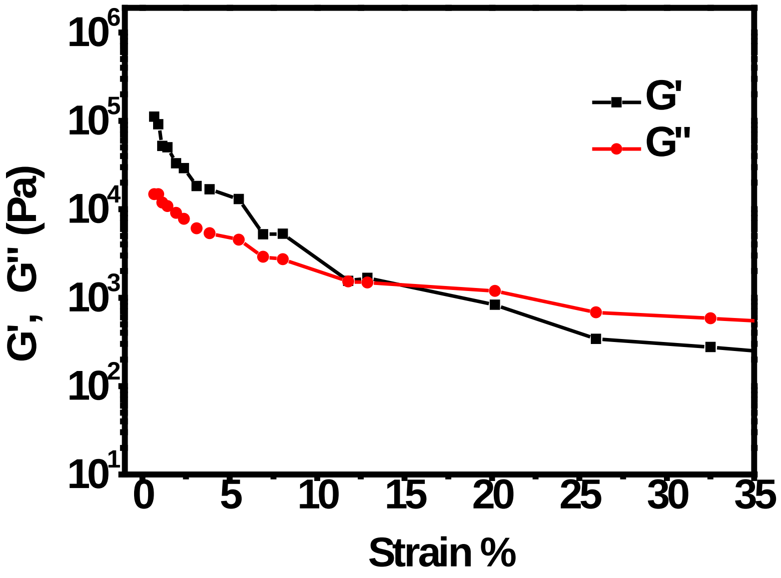

Figure S2. Strain sweep (frequency = 1 Hz) of 2.0% w/v M-GSMG at 25 °C, which revealed the shear thinning characteristics of the M-GSMG with a yield strain of 11.8%.

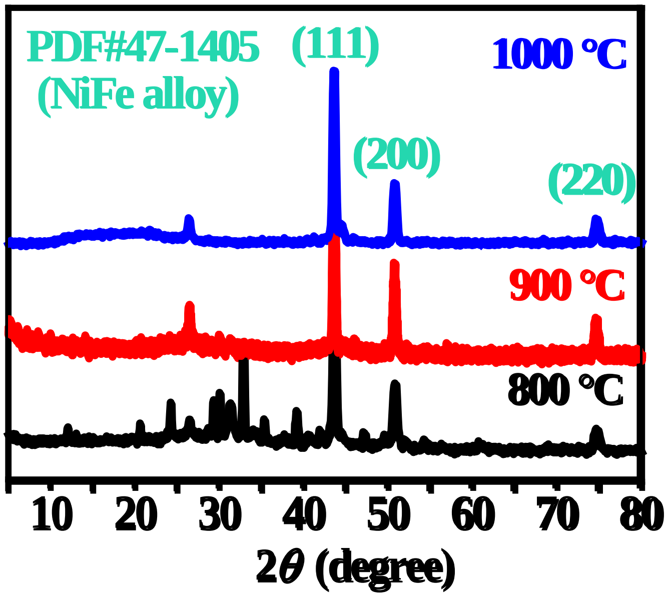

Figure S3. XRD patterns of NiFe/B,N-CNFs obtained under different pyrolysis temperatures.

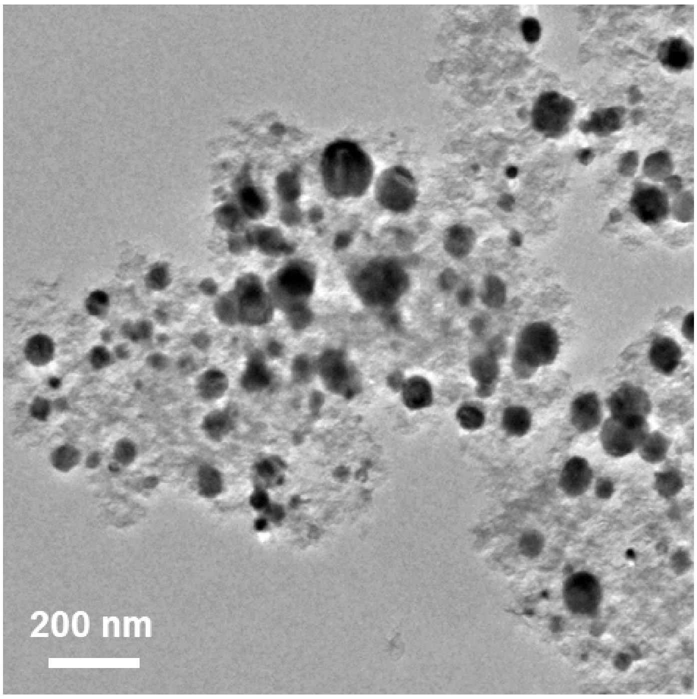

Figure S4. TEM image of NiFe/B,N-CNFs.

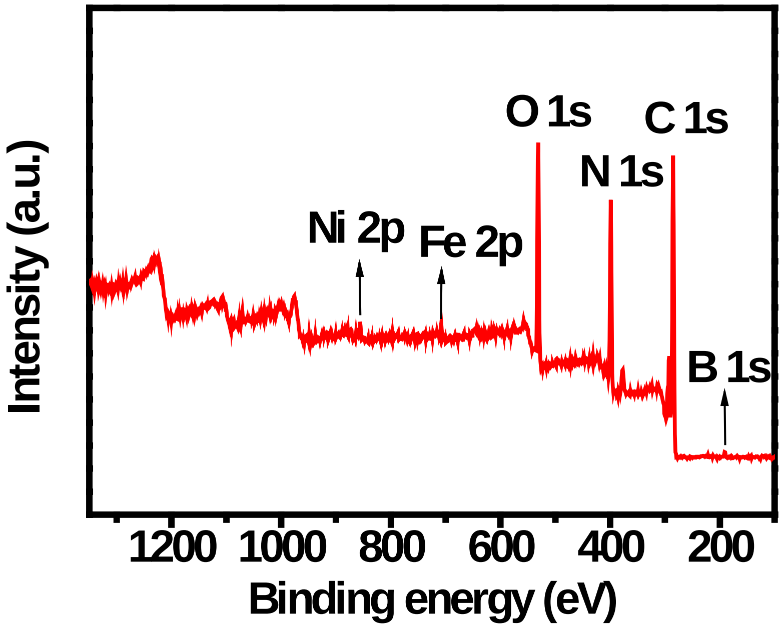

Figure S5. XPS spectrum of the NiFe/B,N-CNFs catalyst.

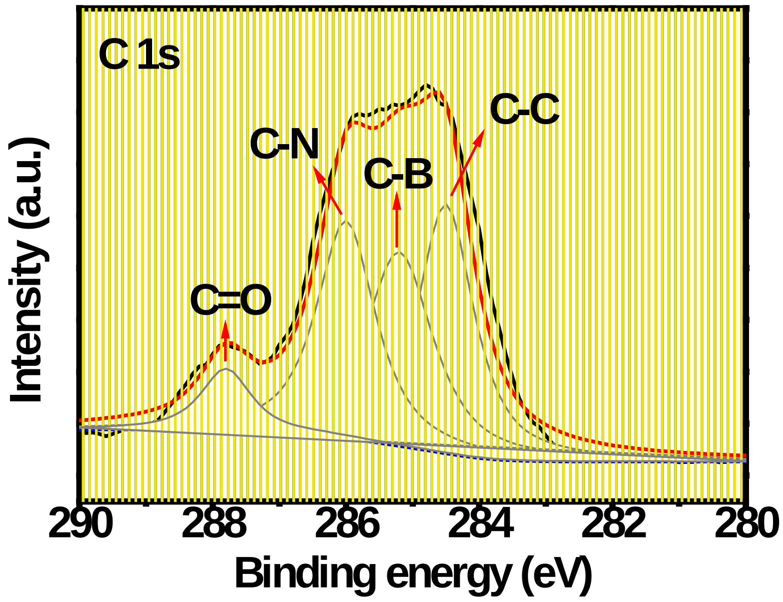

Figure S6. High-resolution XPS spectrum of C 1s core levels in NiFe/B,N-CNFs catalyst.

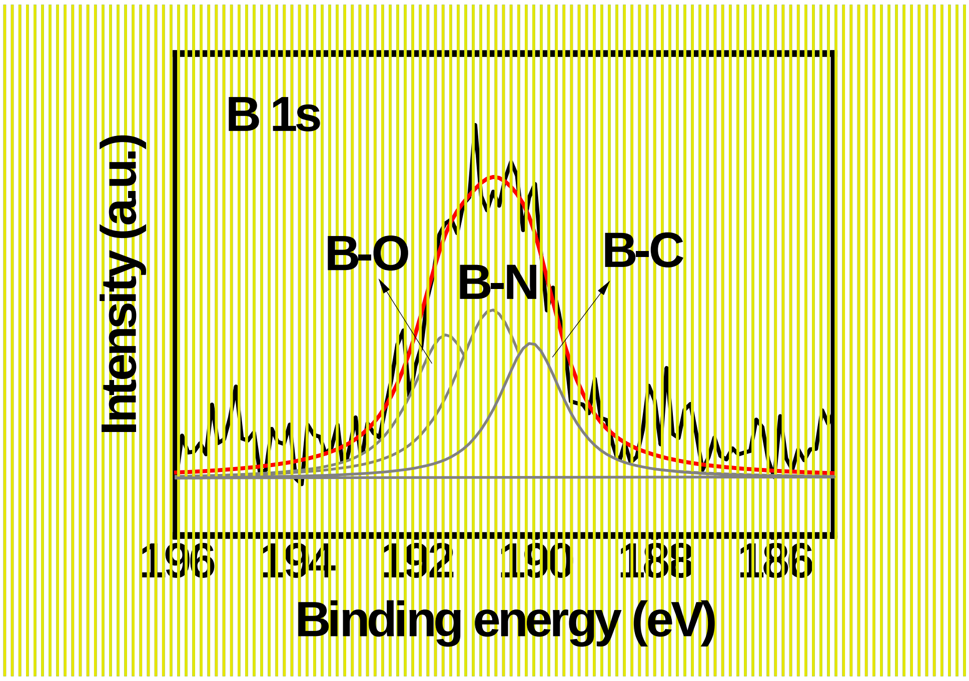

Figure S7. High-resolution XPS spectrum of B 1s core levels in NiFe/B,N-CNFs catalyst.

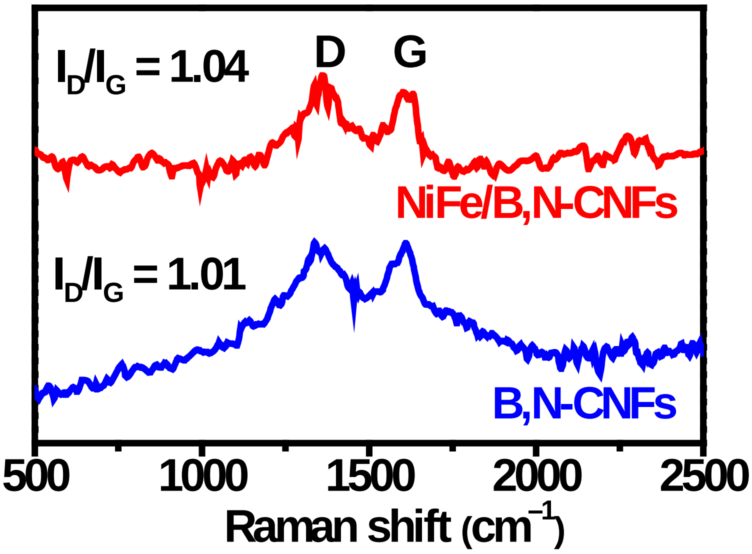

Figure S8. Raman spectra of NiFe/B,N-CNFs and B,N-CNFs.

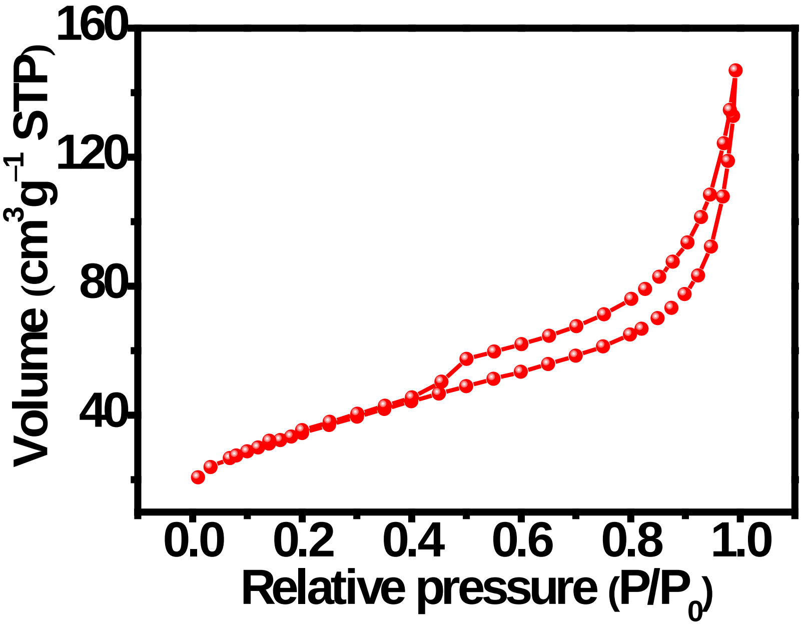

Figure S9. N_2 adsorption–desorption isotherm of NiFe/B,N-CNFs.

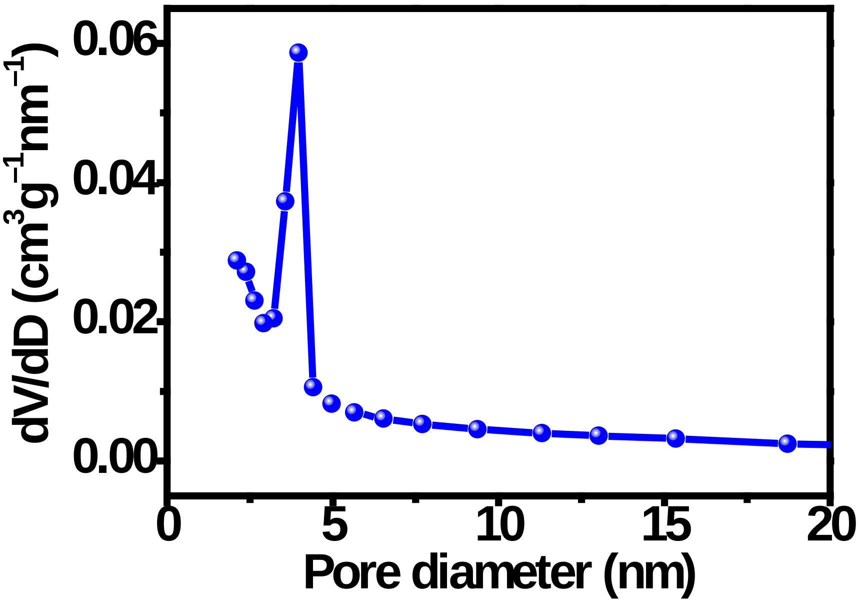

Figure S10. Pore size distribution of the as-synthesized NiFe/B,N-CNFs.

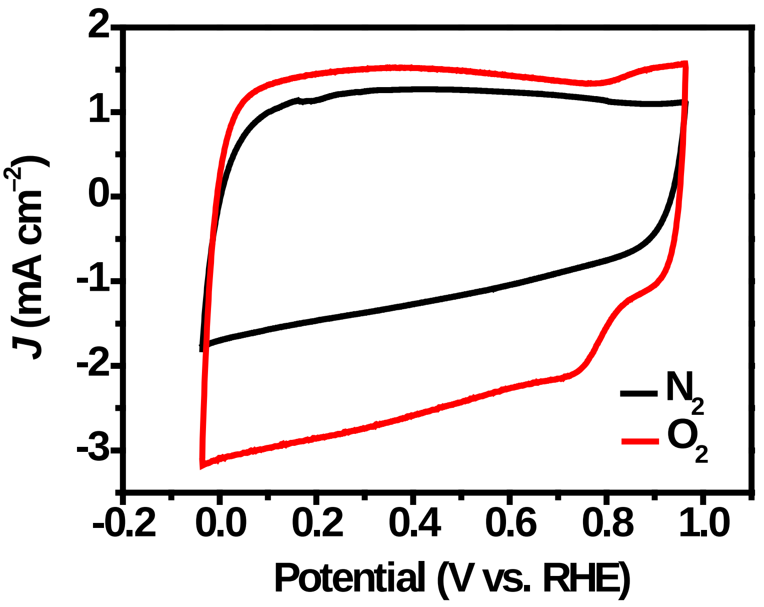

Figure S11. CV curves of NiFe/B,N-CNFs in N_2-/O_2-saturated 0.1 M KOH solution (Scan rate: 100 mV s^{-1}).

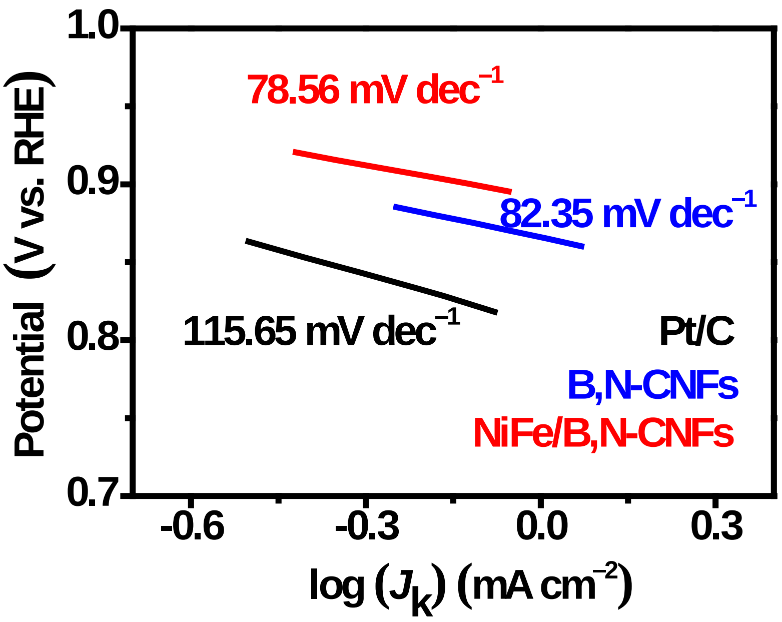

Figure S12. Tafel plots for different catalysts, determined by mass-transport correction of the 1600 rpm rotating disk electrode data.

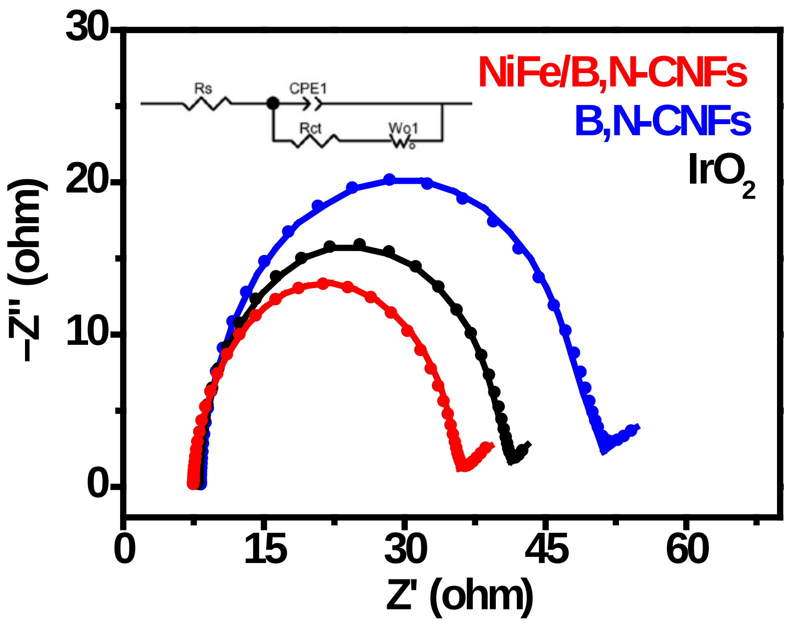

Figure S13. EIS spectra of different catalysts recorded at a constant potential of 1.75 V (vs. RHE).

Table S1. Charge transfer resistance (R_{ct}) of B,N-CNFs, NiFe/B,N-CNFs, and IrO_2 for OER.

**Sample**	**B,N-CNFs**	**NiFe/B,N-CNFs**	**IrO_2**
**R_{ct} (Ω)**	44.95	19.87	27.86

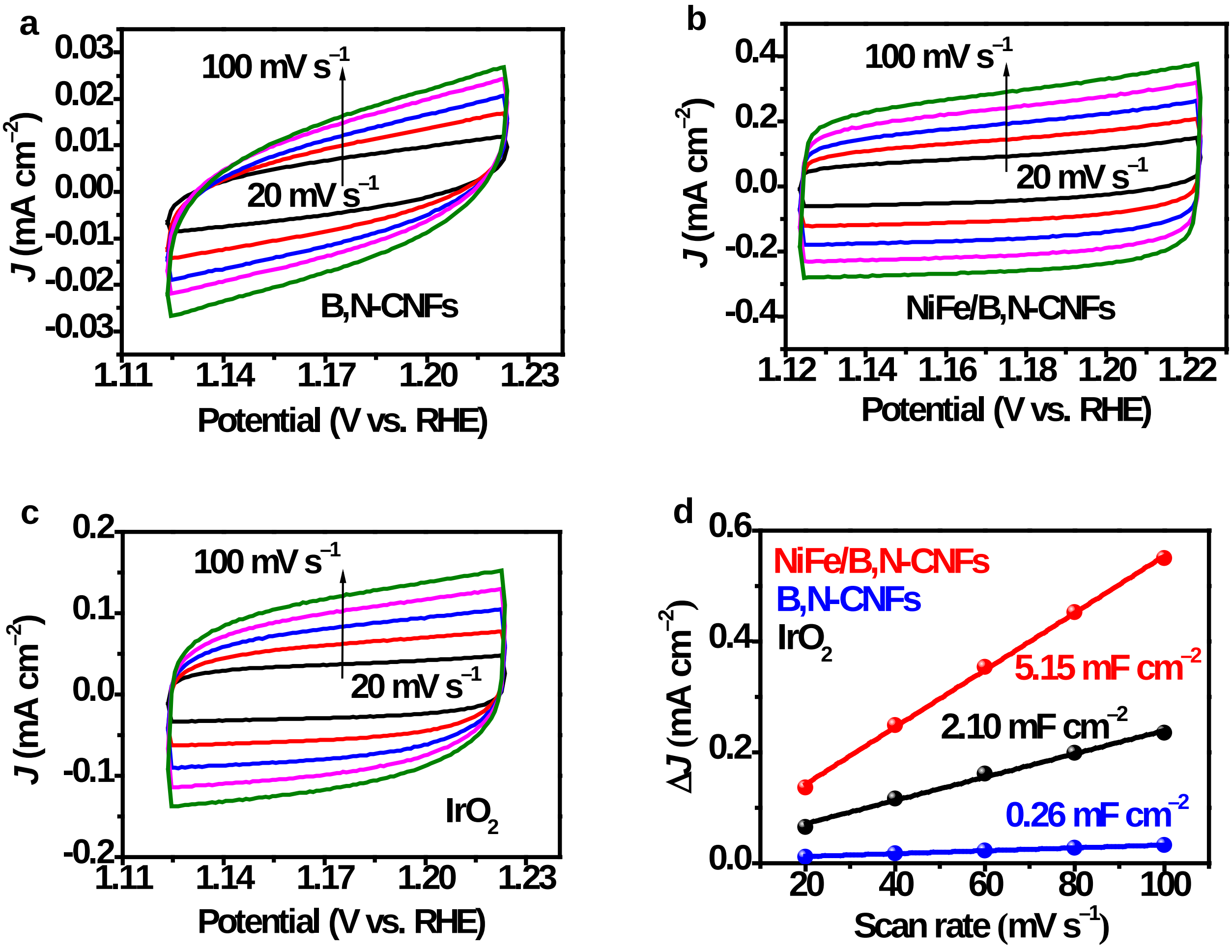

Figure S14. (a-c) Typical CV curves of different catalysts at different scan rates ranging from 20 to 100 mV s^{-1} in 1.0 M KOH solution. (d) Plots of ΔJ versus scan rate of different catalysts measured in 1.0 M KOH solution with GC electrode.

Table S2. Comparison of the catalytic activities of the reported composite electrocatalysts.

**Bifunctional catalyts**	**$E_{1/2}$ (V)**	**$E_{j=10}$ (V)**	**ΔE (V)**	**Refs.**
**NiFe/B,N-CNFs**	**0.84**	**1.52**	**0.68**	**This work**
OLC/Co-N-C	0.855	1.574	0.719	1
FeP/Fe_2O_3@NPCA	0.838	1.632	0.794	2
Fe/N-G-SAC	0.89	1.60	0.710	3
Ni,N codoped np-graphene	0.73	1.50	0.77	4
Pd/FeCo	0.85	1.55	0.70	5
HHPC	0.78	1.58	0.80	6
$NiFe_3$@NC	0.86	1.507	0.647	7
$Ni_{66}Fe_{34}$-NC	0.85	1.697	0.847	8
H-Co@FeCo/N/C	0.91	1.61	0.70	9
$NiPS_3$-xSex NSs	0.75	1.48	0.73	10
CNT@SAC-Co/NCP	0.87	1.61	0.74	11
CoNi-N-CNFs	0.823	1.469	0.636	12
NiFe@NBCNT	0.83	1.425	0.595	13
NiFe@N-CFs	0.82	1.53	0.71	14

Section S3 Electrochemical performance of the omnidirectionally stretchable ZAB at room temperature

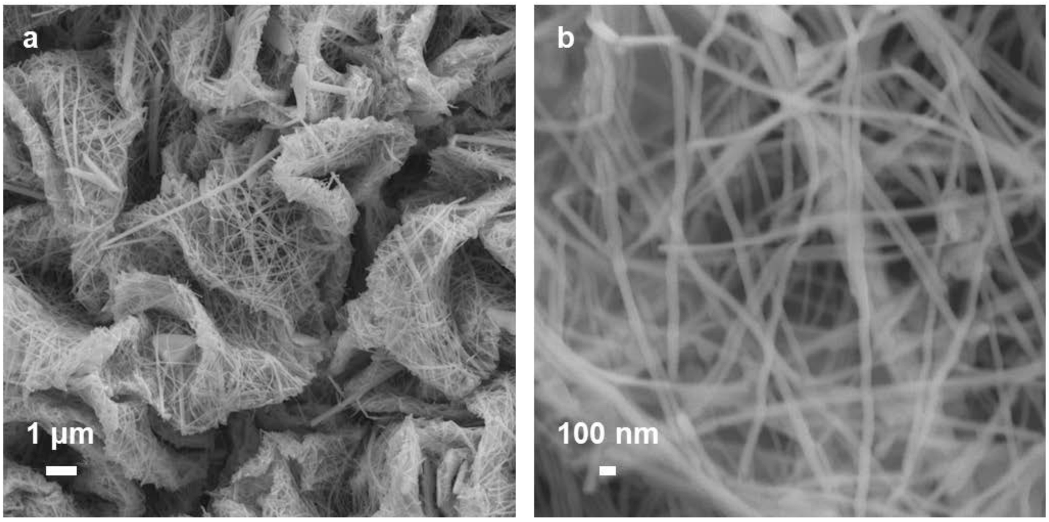

Figure S15. SEM images of Ag NWs on the VHB elastomer.

Table S3. The sheet resistance of the initial and 1000% stretched VHB + Ag NWs current collector.

	**Sheet resistance (Ω sq^{-1})**	
	**Initial**	**1000% stretched**
**1**	0.93	5.17
**2**	0.69	4.90
**3**	1.07	4.38
**4**	0.81	2.53
**5**	0.89	5.50
**6**	1.26	4.57
**7**	0.92	4.25
**8**	1.05	3.28
**9**	0.93	3.30
**10**	0.71	2.81
**Average**	0.93	4.07

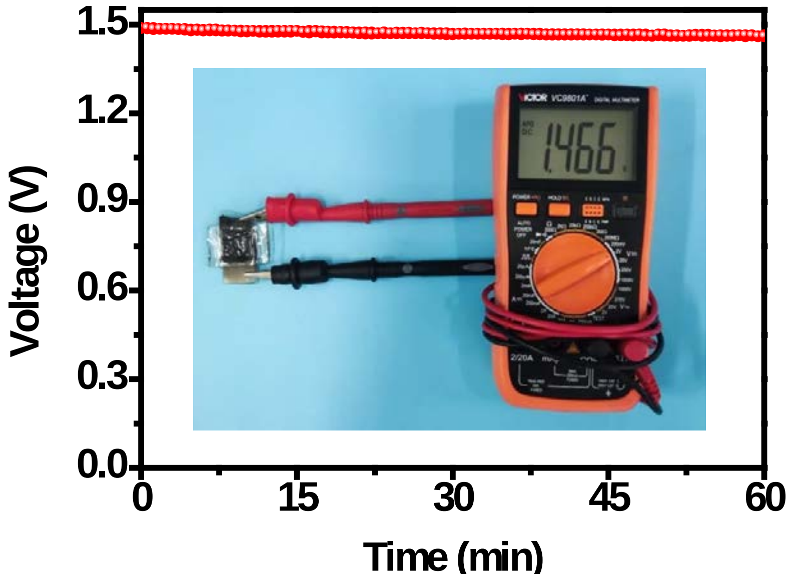

Figure S16. Open circuit voltage of the ZAB at room temperature. Inset is the experimental site photograph.

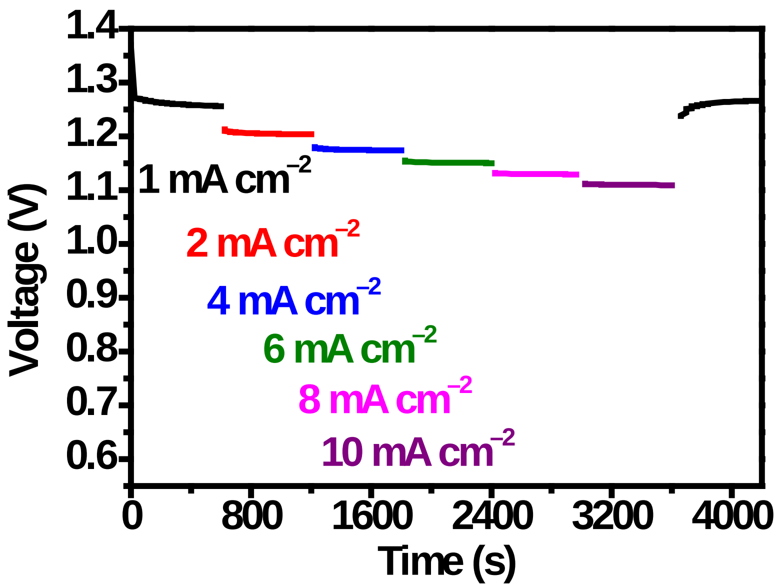

Figure S17. Rate discharge properties of the ZAB at room temperature.

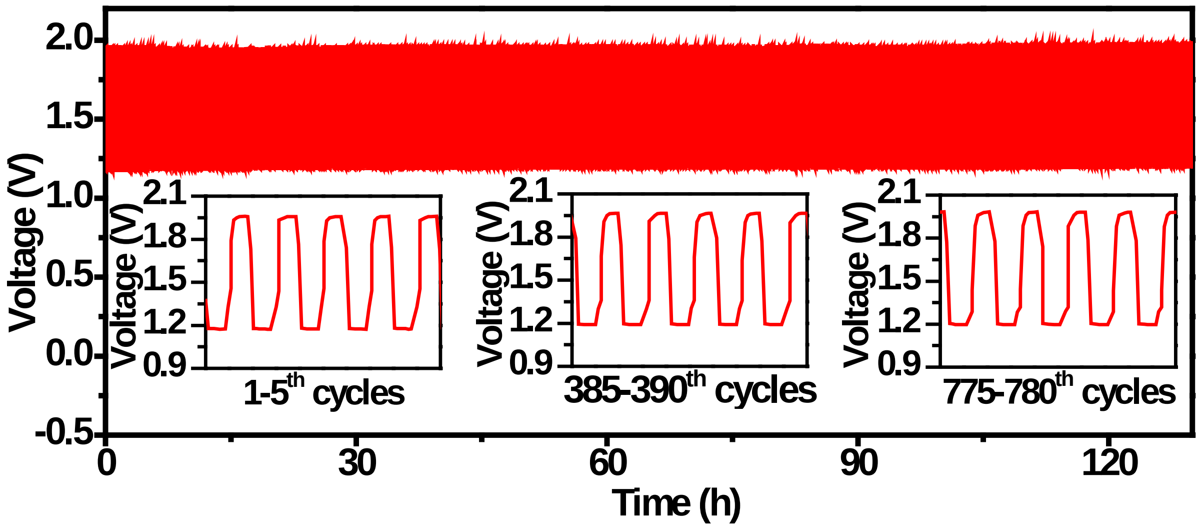

Figure S18. Galvanostatic cycling curves of the ZAB under a current density of 2 mA cm^{-2} at room temperature. Inset: magnified plots of the galvanostatic discharge and charge curves at different cycles.

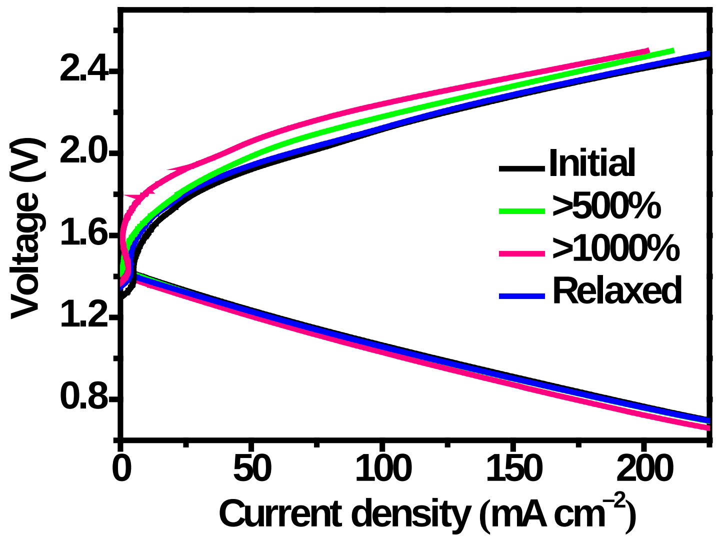

Figure S19. Polarization curves of the ZAB under various areal strains at room temperature.

Section S4 Environmental adaptability of the omnidirectionally stretchable ZAB

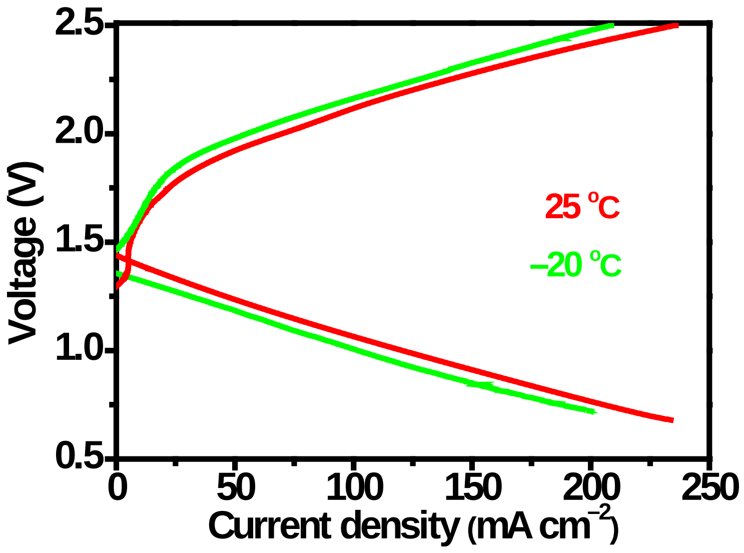

Figure S20. Charge and discharge polarization curves of the ZAB without mechanical strain at 25 and –20 °C, respectively.

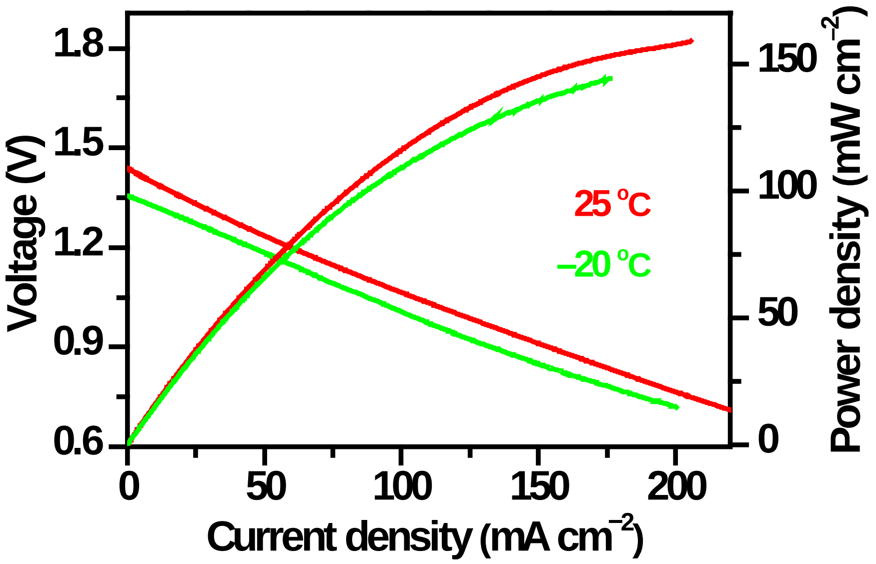

Figure S21. Discharge and power density curves of the ZAB without mechanical strain at 25 and –20 °C, respectively.

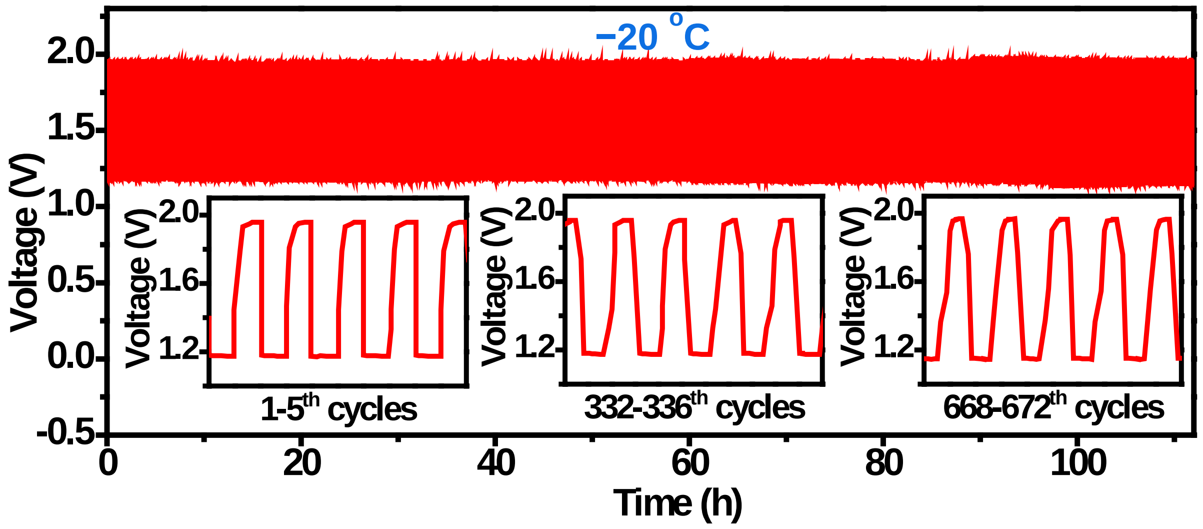

Figure S22. Galvanostatic cycling curves of the ZAB at –20 °C under a current density of 2 mA cm^{-2} . Inset: magnified plots of the galvanostatic discharge and charge curves at different cycles.

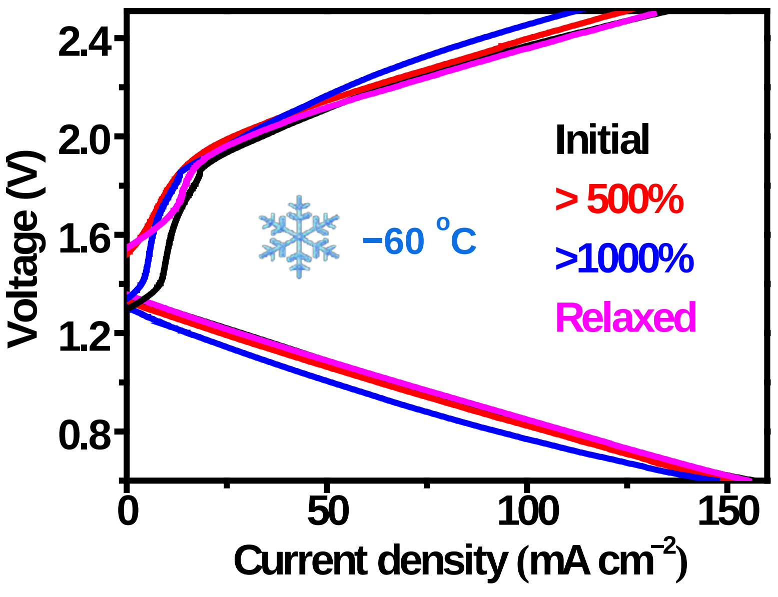

Figure S23. Polarization curves of the ZAB under various areal strains at –60 °C.

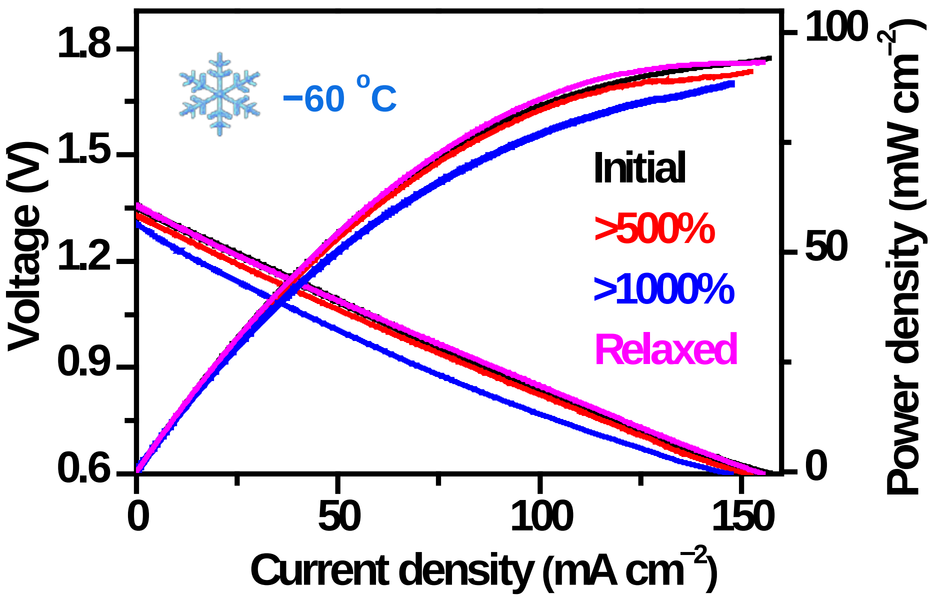

Figure S24. Discharge and power density curves of the ZAB under various areal strains at –60 °C.

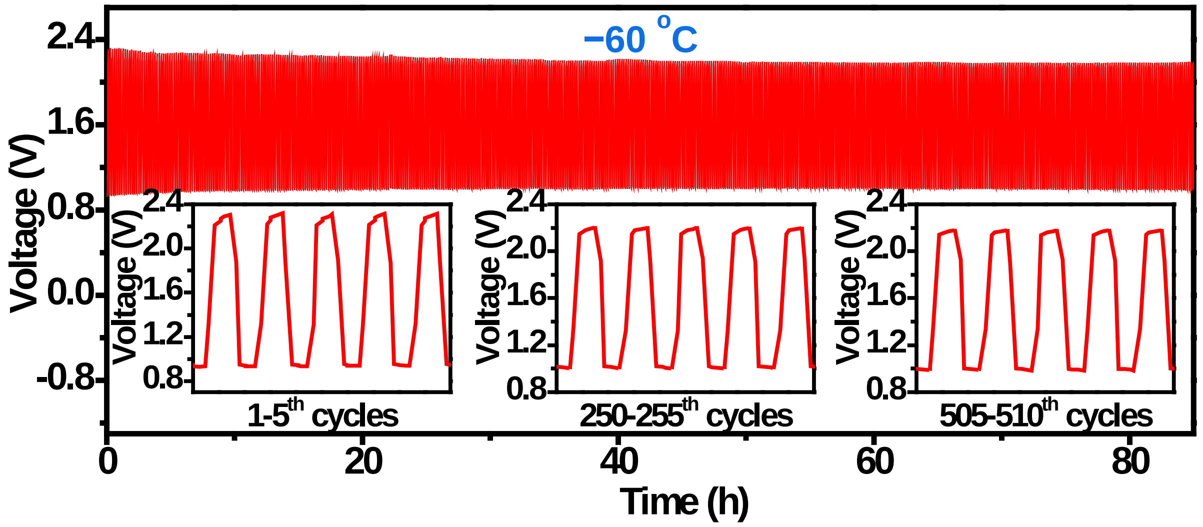

Figure S25. Galvanostatic cycling curves of the ZAB at –60 °C under a current density of 2 mA cm^{-2}. Inset: magnified plots of the galvanostatic discharge and charge curves at different cycles.

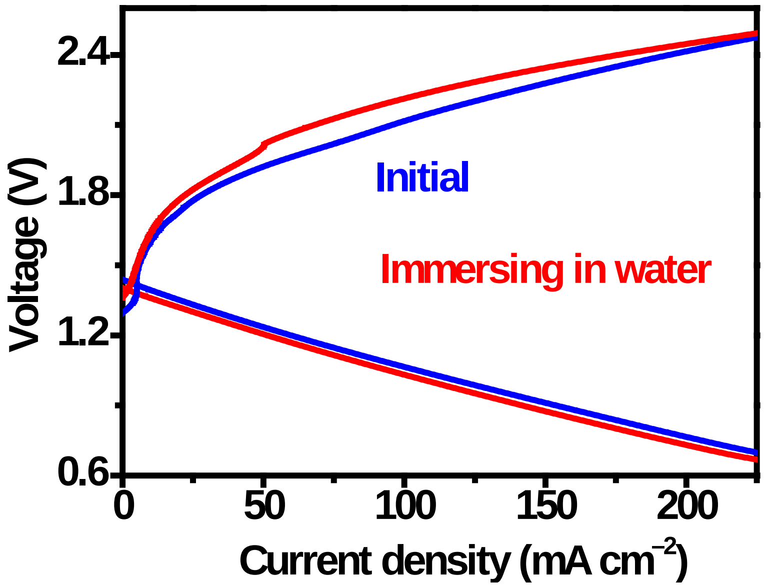

Figure S26. Polarization curves of the ZAB before and after fully immersed in water for 3 h obtained *via in situ* electrochemical test.

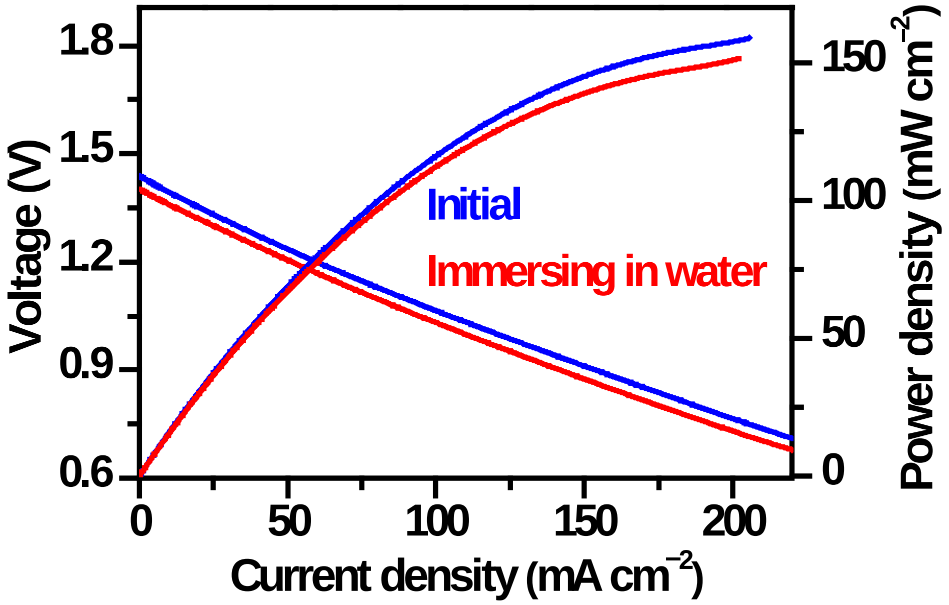

Figure S27. Discharge and power density curves of the ZAB before and after fully immersed in water for 3 h obtained *via in situ* electrochemical test.

Table S4. Comparison of flexible Zn-air batteries reported in the literature.

Type of flexible strategy	Deformability	Power density (mW cm^{-2})	Open-circuit potential (V)	Performance retention	Waterproof performance	Temperature tolerance (°C)	Refs.
**Anode and electrolyte: intrinsic**<br>**Cathode: wavy structure**	**Omnidirectional tension**<br>**Areal strain >1000%**	**159.0**	**1.47**	**87% after 10000 cycles at 400% strain**	**Rinsing >5 h**<br>**Immersing >3 h**	**–60**	**This work**
Wavy coiled spring	Uniaxial tension >1000%	165.3	1.47	-----	Rinsing >25 min<br>Immersing >2 h	–50	15
Wavy coiled spring	Uniaxial tension 800%	108.6	1.48	-----	Immersing 2 h (waterproof treatment)	-----	16
Bridge-island	Uniaxial tension 125%	-----	1.35	-----	-----	-----	17
Sandwich-type	Bending, folding, twisting	160.0	1.49	-----	-----	–20	18

(Continued)

Type of flexible strategy	Deformability	Power density (mW cm^{-2})	Open-circuit potential (V)	Performance retention	Waterproof performance	Temperature tolerance (°C)	Refs.
Cable-type	Bending, knotting	78.6	~1.4	-----	-----	-----	19
Pouch	Bending, folding	-----	~1.5 V	over 500 bending cycles	-----	–20	20
Sandwich-type and Cable-type	Bending	50	1.45	-----	-----	–40	21
Sandwich-type	Bending, twisting, folding	128.8	1.47	-----	-----	–30	22
Sandwich-type	Bending	11.8	1.42	-----	-----	–20	23
Sandwich-type	Bending	73.9	1.35	-----	-----	–20	24

Section S5 References

[1] Z. Liang, N. Kong, C. Yang, W. Zhang, H. Zheng, H. Lin, R. Cao, *Angew. Chem. Int. Ed.* **2021**, *60*, 12759.

[2] K. Wu, L. Zhang, Y. Yuan, L. Zhong, Z. Chen, X. Chi, H. Lu, Z. Chen, R. Zou, T. Li, C. Jiang, Y. Chen, X. Peng, J. Lu, *Adv. Mater.* **2020**, *32*, 2002292.

[3] M. Xiao, Z. Xing, Z. Jin, C. Liu, J. Ge, J. Zhu, Y. Wang, X. Zhao, Z. Chen, *Adv. Mater.* **2020**, *32*, 2004900.

[4] H. Qiu, P. Du, K. Hu, J. Gao, H. Li, P. Liu, T. Ina, K. Ohara, Y. Ito, M. Chen, *Adv. Mater.* **2019**, *31*, 1900843.

[5] F. Pan, Z. Li, Z. Yang, Q. Ma, M. Wang, H. Wang, M. Olszta, G. Wang, Z. Feng, Y. Du, Y. Yang, *Adv. Energy Mater.* **2021**, *11*, 2002204.

[6] X. Xiao, X. Li, Z. Wang, G. Yan, H. Guo, Q. Hu, L. Li, Y. Liu, J. Wang, *Appl. Catal. B Environ.* **2020**, *265*, 118603.

[7] D. Chen, J. Zhu, X. Mu, R. Cheng, W. Li, S. Liu, Z. Pu, C. Lin, S. Mu, *Appl. Catal. B Environ.* **2020**, *268*, 118729.

[8] M. Ma, A. Kumar, D. Wang, Y. Wang, Y. Jia, Y. Zhang, G. Zhang, Z. Yan, X. Sun, *Appl. Catal. B Environ.* **2020**, *274*, 119091.

[9] Y. Wu, X. Wu, T. Tu, P. Zhang, J. Li, Y. Zhou, L. Huang, S. Sun, *Appl. Catal. B Environ.* **2020**, *278*, 119259.

[10] J. Song, S. Qiu, F. Hu, Y. Ding, S. Han, L. Li, H. Chen, X. Han, C. Sun, S. Peng, *Adv. Funct. Mater.* **2021**, *31*, 2100618.

[11] J. Li, Y. Meng, L. Zhang, G. Li, Z. Shi, P. Hou, C. Liu, H. Cheng, M. Shao, *Adv. Funct. Mater.* **2021**, *31*, 2103360.

[12] H. Li, G. Wu, G. Cheng, Y. Shuai, S. Liu, Y. Liu, *J. Alloys Compd.* **2021**, *888*, 161588.

[13] D. Bin, B. Yang, C. Li, Y. Liu, X. Zhang, Y. Wang, Y. Xia, *ACS Appl. Mater. Interfaces* **2018**, *10*, 26178−26187

[14] Y. Niu, X. Teng, S. Gong, Z. Chen, *J. Mater. Chem. A*, **2020**, *8*, 13725-13734.

[15] C. Gu, X.-Q. Xie, Y. Liang, J. Li, H. Wang, K. Wang, J. Liu, M. Wang, Y. Zhang, M. Li, H.

Kong, C.-S. Liu, *Energy Environ. Sci.* **2021**, *14*, 4451.

[16] L. Ma, S. Chen, D. Wang, Q. Yang, F. Mo, G. Liang, N. Li, H. Zhang, J. A. Zapien, C. Zhi, *Adv. Energy Mater.* **2019**, *9*, 1803046.

[17] S. Qu, Z. Song, J. Liu, Y. Li, Y. Kou, C. Ma, X. Han, Y. Deng, N. Zhao, W. Hu, C. Zhong, *Nano Energy* **2017**, *39*, 101.

[18] Z. Pei, Z. Yuan, C. Wang, S. Zhao, J. Fei, L. Wei, J. Chen, C. Wang, R. Qi, Z. Liu, Y. Chen, *Angew. Chem. Int. Ed.* **2020**, *59*, 4793.

[19] Z. Song, J. Ding, B. Liu, X. Liu, X. Han, Y. Deng, W. Hu, C. Zhong, *Adv. Mater.* **2020**, *32*, 1908127.

[20] S. S. Shinde, J. Y. Jung, N. K. Wagh, C. H. Lee, D.-H. Kim, S.-H. Kim, S. U. Lee, J.-H. Lee, *Nat. Energy* **2021**, *6*, 592.

[21] Y. Zhang, H. Qin, M. Alfred, H. Ke, Y. Cai, Q. Wang, F. Huang, B. Liu, P. Lv, Q. Wei, *Energy Storage Mater.* **2021**, *42*, 88.

[22] Z. Pei, L. Ding, C. Wang, Q. Meng, Z. Yuan, Z. Zhou, S. Zhao, Y. Chen, *Energy Environ. Sci.* **2021**, *14*, 4926.

[23] R. Chen, X. Xu, S. Peng, J. Chen, D. Yu, C. Xiao, Y. Li, Y. Chen, X. Hu, M. Liu, H. Yang, I. Wyman, X. Wu, *ACS Sustain. Chem. Eng.* **2020**, *8*, 11501.

[24] N. Sun, F. Lu, Y. Yu, L. Su, X. Gao, L. Zheng, *ACS Appl. Mater. Interfaces* **2020**, *12*, 11778.
